# Genetic dissection of sorghum [*Sorghum bicolor* (L.) Moench] grain antimicrobial activity against *Clostridium perfringens* and its relationship with grain composition and field performance

**DOI:** 10.3389/fpls.2026.1754365

**Published:** 2026-03-16

**Authors:** Maria Antonella Conti, Carolina Ballén-Taborda, Maggie Leigh Thomas, Kimberly Baskins, William Caughman, Chinwe Happiness Justice-Alucho, Tricia King, Daniel Crozier, William L. Rooney, Mireille Arguelles-Ramos, Xiuping Jiang, Andrew K. Benson, Richard Elmer Boyles

**Affiliations:** 1 Pee Dee Research and Education Center, Clemson University, Florence, SC, United States; 2 Department of Biological Sciences, Clemson University, Clemson, SC, United States; 3 Department of Food Science and Technology and Nebraska Food for Health Center, University of Nebraska, Lincoln, NE, United States; 4 Department of Soil and Crop Science, Texas A&M University, College Station, TX, United States; 5 Department of Animal and Veterinary Sciences, Clemson University, Clemson, SC, United States; 6 Department of Food, Nutrition and Packaging Sciences, Clemson University, Clemson, SC, United States

**Keywords:** antimicrobial activity, clostridium perfringens, foodborne pathogens, functional feed, non-tannin sorghum, QTL mapping

## Abstract

In the poultry industry, there is a growing interest in finding alternatives to synthetic antibiotics to maintain gut health, given the growing problems with antibiotic resistance in several pathogenic species. Antibiotics are especially used to control one of the most widespread and problematic diseases of poultry, necrotic enteritis, which is caused by *Clostridium perfringens*. Sorghum grain is known to have bioactive compounds that can provide antimicrobial (AM) activity, with most of these compounds being phenolics. Condensed tannins are produced by sorghum and known for their potent antioxidant and AM activity; however, they also have antinutritional effects in the poultry gut. Thus, the AM potential of non-tannin sorghum grain against *C. perfringens* was examined. Two distinct analyses were performed on a non-tannin recombinant inbred line (RIL) population to measure AM activity of the grain against *C. perfringens*: 1) a minimum inhibitory concentration (MIC) analysis with phenol extract of the grain, and 2) a qPCR-based approach to measure inhibition of *C. perfringens* in monoculture with *in vitro* enzymatically digested grain. It was found that the RIL population exhibited different levels of AM activity against the bacteria, with some lines having activity comparable to that of tannin sorghums, indicating that genotypes lacking functional *Tan1* and *Tan2* genes maintain a sufficient level of AM activity. Additionally, no negative impact on agronomic performance, grain productivity or quality traits was observed, while positive correlations were found with desirable traits such as grain yield and grain mold resistance. Furthermore, through QTL mapping, novel loci were identified associated with AM activity, with a major-effect QTL on chromosome 1 that was distinct from the location of the pericarp color gene *YELLOWSEED1*. Three other loci were identified on chromosomes 3, 4 and 6, having additive and interaction effects. This suggests that, although the AM activity is controlled by multiple interacting genes, there are some major-effect genes that could be targeted to increase this trait in breeding lines. In this biparental population, 23 RILs possessed optimal haplotypes to serve as AM activity donors for introgressing this trait into sorghum improvement programs.

## 1 Introduction

Intensive animal production systems that routinely utilize antimicrobials to maintain health and productivity have raised concerns surrounding antibiotic resistance and its implications on global human and animal health (Silbergeld et al., 2008). The animal production industry uses 73% of the global antimicrobials sold to treat bacterial infections in animals raised for food (Van Boeckel et al., 2019), and there is evidence that links this practice with the rise of antimicrobial-resistant infections, both in animals and in humans (Ward et al., 2014; De Vries et al., 2018). The poultry industry is an extremely intensive production system given the number of animals raised per area; there is a growing interest in finding alternatives to antibiotics to maintain gut health (Oviedo-Rondón, 2019) and promote well-being and performance. One of the most extended and problematic diseases in the poultry industry is necrotic enteritis (NE). NE is caused by the bacterium *Clostridium perfringens*, which can plague a poultry operation and lead to significant challenges for management of the birds (Moritz et al., 2023). *C. perfringens* is a ubiquitous Gram-positive, anaerobic bacterium that can produce a range of toxins (Songer, 1996). Strains of *C. perfringens* are found frequently as a member of the gut microbiota in otherwise healthy animals and humans (Hatheway, 1990) but generally lack genes encoding most or all of the toxins, whereas more virulent strains carry one or more plasmid-encoded toxin genes (Mehdizadeh Gohari et al., 2021). Modifications of the intestinal environment, including changes to the physicochemical properties and alterations to the microbial composition, facilitate the colonization of pathogenic *C. perfringens* strains in the gut. Colonization of poultry by pathogenic *C. perfringens* strains producing chromosomally-encoded sialidases and the plasmid-encoded NetB toxin can degrade the mucus of the gut and damage the endothelial cells (NetB toxin), leading to NE (Cooper and Songer, 2009; Prescott et al., 2016). NE outbreaks are treated with antimicrobials, but to control subclinical NE, which shows no visible symptoms, antimicrobial growth promoters (AGPs) are generally used (Huyghebaert et al., 2011). However, *C. perfringens* susceptibility to antibiotics has declined over the years. This has been attributed, in part, to the use of subtherapeutic doses of antimicrobials (Olazabal et al., 2005) and the use of AGPs at subinhibitory doses for extended periods of time, ultimately selecting for the emergence of resistance to antimicrobials. Moreover, the establishment of resistant *C. perfringens* strains in poultry farms can cause the transfer of resistant bacteria and their resistance factors from animals to humans (Diaz Carrasco et al., 2016).

Therefore, the reliance on synthetic antimicrobials threatens the sustainability of the animal production industry (Van Boeckel et al., 2019). Implementing alternative solutions, such as the development of nutritionally sufficient feedstuffs with natural antimicrobial (AM) properties, is warranted to combat the continuing rise in antibiotic resistance in animal agriculture (Gyawali and Ibrahim, 2014). Among the feed grains used in poultry diets, sorghum [*Sorghum bicolor* (L.) Moench] is noteworthy for its high levels of bioactive compounds (Bravo, 1998), which have antioxidant, anti-inflammatory, and AM properties (Rao et al., 2018). Sorghum has been measured to have one of the highest levels of phenolic compounds among cereal crops (Awika, 2011; Kang et al., 2016). The content and profile of these compounds differ greatly among sorghum varieties and are a function of genetics that regulate the color of the pericarp and presence (or absence) of a pigmented testa, and environmental conditions during crop growth (Wu et al., 2017). These compounds are constitutively present in the plant and are part of their defense response to environmental stress and pathogen attack. Their content can increase, and some compounds can be produced *de novo* as part of the plant’s immune response (Boddu et al., 2004; Poloni and Schirawski, 2014; Piasecka et al., 2015; Nida et al., 2019). These polyphenols, which may help protect poultry from diseases and regulate their immune responses (Shields et al., 2021), make sorghum a valuable and functional food or feed by conferring health benefits to humans and animals (Ridaura et al., 2013; Singh et al., 2017). Despite their AM and antioxidant properties, some of these compounds can reduce feed intake or negatively affect nutrient digestibility, potentially resulting in reduced weight gains in broilers (Nyachoti et al., 1997). A clear example is condensed tannins, highly polymerized phenolic compounds that possess potent antioxidant properties with potential health benefits (Waniska et al., 1989; Serna-Saldivar, 2016). Despite the health benefits, condensed tannins are also well known for their anti-nutritional effects, where they bind to dietary protein and reduce protein digestibility as well as binding to digestive enzymes, reducing their activity. These activities ultimately inhibit growth rates and lower feed efficiency (Cousins et al., 1981; Serna-Saldivar, 2016). Thus, increasing tannin concentration often decreases sorghum’s feed value. The presence and content of tannins are genetically controlled by two epistatic genes, *Tannin1* (*Tan1* on chromosome 4 at 62.3 Mb, *Sobic.004G280800*) and *Tannin2* (*Tan2* on chromosome 2 at 7.97 Mb, *Sobic.002G076600*) that must both be functional for considerable tannin production in the sorghum grain (Wu et al., 2012, 2019). Loss-of-function at either *Tan1* or *Tan2* leads to non-tannin (or tannin-free) sorghums (Zhang et al., 2024), and naturally occurring mutations in these genes are found in many non-tannin sorghum lines (Wu et al., 2019). Commercial sorghum hybrids in the United States do not contain tannins, and breeding for low-tannin sorghums remains a priority.

Studies have demonstrated that non-tannin sorghum grain can inhibit *C. perfringens* growth with no adverse effects on poultry performance. Shields et al. (2021) observed that grain extracts from diverse sorghum accessions significantly inhibited the growth of *C. perfringens* and a second foodborne pathogen commonly found in poultry, *Salmonella enterica*. While a positive correlation was found between tannin concentration and AM activity for *S. enterica*, no relationship was observed between grain extract tannins and *C. perfringens* inhibition. This study suggested that sorghum possesses additional types of bioactive compounds that provide AM activity (Morris et al., 2013), and this non-tannin sorghum with AM activity could be used as a poultry feed ingredient to support better health. To this end, studies of birds fed sorghum grain had significantly reduced concentrations of *C. perfringens* in the microbiome, and, as a result, these tannin-free sorghums decreased the number of lesions in the poultry gut wall that result from *C. perfringens* infection (Garcia et al., 2013; Moritz et al., 2023). A separate study of the effect of corn replacement by non-tannin sorghum in broiler diets found no adverse effect on birds’ body weight gain, feed conversion ratio, average daily weight gain and average daily feed intake (Puntigam et al., 2020).

Bioactive compounds in the grain can shape the composition and function of the gut microbiome. Yang et al. (2022) investigated this on human gut microbiomes, using sorghum grain and an automated *in vitro* microbiome screening (AiMS), a novel, high-throughput *in vitro* fermentation of pre-digested grain, followed by quantification of microbial taxa abundance for QTL analysis. Digesting the grain *in vitro* with key digestive enzymes and fermenting it with specific bacteria or groups of microbes (microbiome) supports investigating the effect of functional feed ingredients under conditions that better resemble what naturally happens in the gut. The digestion process can release compounds that are not bioavailable when performing single chemical extractions. Further, the fermentation process provides added time for the bacteria to interact with the digested grain, thereby mimicking the diet-microbiome interactions that occur in the large intestine. The study by Yang et al. (2022) identified 26 QTLs across nine sorghum chromosomes associated with variation in microbial abundance, highlighting the quantitative nature of this trait. Additionally, they observed an association between tannin content and shifts in microbiome compositions, with two of the QTLs overlapping with *Tan1* and *Tan2* genes.

Furthermore, when mapping the AM activity of sorghum, total phenols concentration, which was found to be associated with it, a significant SNP was found on chromosome 2, at 7.5Mb, mapping to the *Tan2* locus (Morris et al., 2013; Rhodes et al., 2017; Shields et al., 2021). Shields et al. (2021) also identified loci associated with AM activity on chromosomes 2 (at 8.9Mb), 4 (at 64Mb), and 10 (at 56 Mb), different from *Tan1* and *Tan2*. These findings suggest that AM activity in grain sorghum is controlled by multiple genetic factors and does not depend solely on condensed tannins. Therefore, studies have shown that phenolic compounds of sorghum grain are involved in AM effects and, although condensed tannins play an important role, genetic mapping demonstrated that there is AM activity that is not driven by *Tan1* or *Tan2*. *In vivo* studies in poultry have demonstrated beneficial effects on the gut of sorghum inclusion in feed ration (Garcia et al., 2013; Moritz et al., 2023), but research focused on the AM activity of non-tannin sorghum and its relationship with field performance or grain quality is lacking.

In this study, we investigated the AM activity of non-tannin sorghum grain against the bacterium *C. perfringens* and the effect of the AM trait on agronomic performance and grain composition. An established recombinant inbred line (RIL) population was leveraged due to known segregation of compositional traits that could influence AM activity, as detailed below in the parent descriptions. The segregating F_6_ RIL population was field-grown and characterized for yield, yield-related traits, agronomic and disease resistance traits. Two different techniques were employed to quantify the AM activity of the grain: a minimum inhibitory concentration (MIC) analysis using phenol extraction and an *in vitro* analysis of growth inhibition in monoculture of *C. perfringens* using grain pre-digested with key poultry digestive enzymes to resemble *in vivo* gut conditions. Phenotypic data collection was used to (1) examine trait relationships to understand potential negative linkage with the AM trait and (2) conduct a QTL analysis for identifying genetic regions associated with the AM trait.

## 2 Materials and methods

### 2.1 Plant material, experimental design, and field trial management

An established population of 189 RILs from the cross between Tx2911 and P850029 was provided by Dr. William Rooney of the Texas A&M University sorghum breeding program. Both lack the presence of a testa, meaning they have no tannins; thus, the RIL population is tannin-free. Tx2911 has a red pericarp (*RRYY*), high phenolic content, carries the intensifier gene (Esele et al., 1995), which makes the pericarp color look brighter, and is tolerant to sorghum grain mold (SGM) and grain weathering. Conversely, P850029 has a white pericarp color (*RRyy*), is a high lysine line, and possesses a highly digestible protein trait (Weaver et al., 1998) that has been shown to increase grain mold susceptibility.

The F_6_ RILs and the two parents (191 total genotypes) were planted in 2023 in Florence, South Carolina, USA, at the Clemson University Pee Dee Research and Education Center. The experiment was planted on 17 May in an alpha-lattice design with two replicates (complete blocks) and a sub-blocking of 10 incomplete blocks each to account for field variability. Each plot consisted of two rows, 6.1 m in length and spaced 0.762 m apart, with 100 seeds planted per row, resulting in a seeding rate of 215,000 seeds ha^-1^.

Pre-plant fertilizer containing muriate of potash (0-0-60), sulfate of potash magnesia (0-0-22-22S), and monoammonium phosphate (11-52-0) was applied two weeks before planting based on soil sample recommendations. Supplemental nitrogen (UAN) using split side-dress applications of 67 kg ha^-1^ and 45 kg ha^-1^ was applied four and six weeks after planting, respectively. A pre-emergent of Medal II ATZ (S-metolachlor and atrazine) at 4.7 l ha^-1^ and glyphosate (1.75 l ha^-1^) was applied immediately after planting, and a post-emergent of Warrant (4.7 l ha^-1^) and atrazine (2.3 l ha^-1^) was applied four weeks after planting. Prevathon (1.5 l ha^-1^) and Sivanto Prime (0.5 l ha^-1^) were applied approximately two months after planting to control for headworms and sorghum aphids, respectively.

### 2.2 Field data collection

The number of plants per plot (Stand Count) was counted 30 days after planting. Days to anthesis (DTA) were recorded every other day as the number of days after planting when 50% of the plants in the plot reached mid-anthesis (visible anthers at or past the middle of the panicle). Plant height (cm) was taken by averaging two representative plants from each plot, measured from the plant base to the apex of the main panicle shortly after physiological maturity. The total number of fully developed panicles per plot was counted at maturity and recorded as panicles per plot (PPP). Pericarp color was determined visually on a plot basis and annotated as W (white), R (red), bR (bright red), or their combination (*i.e.*, RW, bR-W, or bR-R) if the plot was clearly segregating.

Each plot was also evaluated for two naturally present diseases: Anthracnose (caused by *Colletotrichum sublineola*) and SGM (caused by *Fusarium* spp. in association with other fungal species; see Ackerman et al. (2021) for specific details). The anthracnose rating was taken during grain filling (23 August) using a 0–9 visual scale assessing the percentage of disease (leaf area affected) on the plot, where 0 = no symptoms and 9 = 90% or more leaf area destroyed (Bock and Jeger, 2002). SGM rating was taken at physiological maturity (11 September) using visual panicle grain mold severity ratings (PGMSR) at a plot level, assessing the percentage of infected grain in the plot using a 0–9 rating scale where 0 = <10% of infected grains in the plot and 9 = 90% or more of infected grains.

### 2.3 Harvest and post-harvest

Five consecutive primary panicles from the middle of each row of each plot (ten total panicles per plot) were hand-harvested at physiological maturity to obtain a pure seed source for downstream analyses. For RW or bR-W plots, five panicles from each color were harvested, processed and analyzed separately. The plots were then combine-harvested over two consecutive days, and grain yield was calculated based on plot weight and adjusted for percent moisture. Hand-harvested panicles were individually threshed, grain weights were recorded for each panicle, and grain from individual panicles was then bulked together per plot. One thousand grains were counted using a Model U seed counter (International Marketing and Design), and thousand-grain weight (TGW, g) per plot was recorded. These weights were then added to each plot’s combine yield to get a total grain yield per plot (kg ha^-1^).

A grain sample of 50 g from each plot was ground to 1 mm particle size with a CT 193 Cyclotec Sample Mill (FOSS North America) for compositional analysis via near-infrared spectroscopy (NIRS). To assess antimicrobial (AM) activity against *C. perfringens*, sub-samples of 500 mg and 5 g were used for a minimum inhibitory concentration (MIC) and an *in vitro* digestion with qPCR analysis, respectively.

### 2.4 Grain compositional data

Compositional data were collected from each ground grain sample by near-infrared spectroscopy (NIRS), performed with a DA7250 NIR analyzer (Perten Instruments) using existing calibrations (Boyles et al., 2017). The NIRS provides quantitative estimates of several grain macro- and micronutrients. Seven traits of interest were measured, including crude protein (%), starch (% dry basis), crude fat (%), gross energy (cal/g), *in vitro* starch digestibility (IVSD, %), amylose (% starch) and amylopectin (% starch).

A total of 397 samples were analyzed with NIRS, 393 of which correspond to RILs and four correspond to the parents. Three samples had insufficient grain for analysis; therefore, three RILs (CS145, CS190 and CS232) only had one biological replicate for compositional traits.

### 2.5 Phenolic extractions and minimum inhibitory concentration analysis

Phenolic compounds were extracted from each ground grain sample using an acetone extraction. For each sample, 5 ml of 70% acetone was added to 500 mg of ground grain, which was then shaken on an orbital shaker for 2 hours at a speed of 200 rpm and stored at -20°C overnight. The next day, samples were centrifuged at 2970 x g for 10 minutes at 4°C, and the supernatant was decanted into new tubes. An additional 5 ml of 70% acetone was added to the pellet, which was then shaken (for 2 hours at a speed of 200 rpm) and centrifuged (at 2970 x g for 10 minutes at 4°C). This supernatant was added to the previously collected supernatant. Samples were then dried completely under nitrogen evaporation in a heating block at 30°C with a 5–7 LPM flow under a hood. Once samples were dried, extract weights were recorded, and 1,000 µl of 40% DMSO was added to each tube and vortexed to completely suspend the dried extract.

The extract concentration of each sample varied due to differences in the extraction efficiency and grain heterogeneity, with concentrations ranging from 14.9 mg ml^-1^ – 32.9 mg ml^-1^ (after removing statistical outliers). Moreover, taking the exact weight of all the extract samples and resuspending them in an equal volume of DMSO would have sufficed; however, the limited amount of sample extract was a major limitation. To account for the variation in the concentrations during MIC, the extracts were serially diluted in double-fold increments, which normalized the inherent variation in sample concentrations to ensure the MIC reflected the true antimicrobial activity of each extract, avoiding bias from starting concentration differences. The double-fold serial dilutions of the extracts were made using Brucella blood broth by adding equal volumes of the extract to equal volumes of broth sequentially to obtain 2x, 4x, 8x, 16x, 32x and 64x dilutions of the extracts. The MIC of each sample was determined by the microbroth dilution method following the Clinical Laboratory Standard Institute (CLSI) by dispensing 100 µl of each dilution into a 96-well plate.

*Clostridium perfringens* (strain CP6), provided by Southern Poultry Research Group (SPRG) (Nicholson, Georgia, USA), was grown anaerobically at 37°C to achieve logarithmic growth, after which it was centrifuged at 4000 x g for 5 minutes. The pellet cells were resuspended in 0.85% sterile saline solution, and the optical density was adjusted to approximately 0.5 at 600 nm, equivalent to 1.0 X 10^8^ CFU ml^-1^. Following 10-fold serial dilutions, 10 µl of 10^6^ CFU ml^-1^ of the CP6 was added to each well to obtain a final bacterial density of 10^5^ CFU ml^-1^ in each well. This was done in triplicate, with an additional replicate containing the sample dilutions and the broth only as the sample sterility control. Growth control wells contained only the broth and CP6, while antibiotic control was provided using different concentrations of tetracycline prepared from a 64 µg ml^-1^ stock concentration. Filter-sterilized DMSO (40%) inoculated with CP6 was used as a DMSO control. All the plates were incubated anaerobically at 37°C for 24 hours, after which MIC was recorded as the wells containing the least concentration of the extract that was able to inhibit the growth of CP6, as evidenced by the presence of turbidity and cell pellets in the wells. The results were validated by plating out samples from each well on brain heart infusion agar and incubating anaerobically for 24 hours at 37°C to determine isolate viability. The lowest concentrations of the samples with no colonies or colonies fewer than 30 CFU ml^-1^ were considered the MIC value.

A total of 397 samples were analyzed, comprising 393 RILs and four that corresponded to the parents. Three samples failed to produce enough grain for analysis, with three RILs (CS145, CS190 and CS232) having only one datapoint. An additional two samples, one of corn and one of high-tannin sorghum, were added for reference.

### 2.6 *In vitro* enzymatic digestion and qPCR

A total of 398 samples were analyzed, 394 of which corresponded to RILs and four to the parents. Two samples had insufficient grain for analysis; therefore, two RILs (CS190 and CS232) only had one biological replicate. An additional two samples, one of corn and one of high-tannin sorghum, were added for reference. For each sorghum line, 2.5 g of milled seed was mixed with 30 ml of water in a 50 ml Falcon tube for 20 min at 200 rpm on an orbital shaker until fully dispersed. Tubes containing the slurries were immersed in boiling water for 20 min with constant agitation. The slurries were then placed on an orbital shaker (200 rpm) for 40 min. The pH was adjusted to 2.5 with 1 M HCl, followed by the addition of 1 ml of 10% (wt/vol) pepsin (P7000; Sigma, St. Louis, MO) in 50 mM HCl. The slurry was then incubated on an orbital shaker (200 rpm) at 37 °C for 60 min. The pH was then adjusted to 6.9 with 0.5 M NaHCO3, followed by addition of 5 ml of 12.5% (wt/vol) porcine pancreatin (P7545; Sigma, St. Louis, MO) in 0.1 M sodium maleate buffer, and 0.2 ml of *Aspergillus niger* amyloglucosidase (E-AMGDF, 3,260 U/ml, Megazyme). The slurry was incubated for 6 h at 37 °C with orbital shaking at 200 rpm. Following digestion, the material was transferred to dialysis tubing (molecular weight cutoff of 2000 Dalton) and dialyzed against distilled water for 72 hours at 4 °C with a water change every 12 h. The dialyzed samples were then lyophilized and stored at -80 °C for further use.

*In vitro* batch fermentations were performed inside an anaerobic chamber. The digested and lyophilized seed components were resuspended in 30 ml of ddH20 and 575 µl of the resuspended sample was added to a 1 ml-deep well (96-well plate) with 65 µl Brain Heart Infusion broth (BHI). The plates were then placed into the anaerobic chamber and reduced at 4 °C in the with anerobic gas generator (Mitsubishi™ AnaeroPack-Anaero, Japan) for 3 days. After reduction, each well was inoculated with 100 μL of a master culture (previously quantified and stored at -80 °C in 15% glycerol) of *C. perfringens* CP6. This strain is a live-attenuated strain used for vaccine research (Thompson et al., 2006) and was obtained from the University of Georgia Veterinary Diagnostic Center through a materials transfer agreement. After inoculation, the *in vitro* fermentations were then incubated anaerobically at 37 °C for 8 h. Growth was terminated by centrifugation at 4000 g for 10 min and the pellets were stored at -80 °C until further processing. The terminal bacterial growth at the end of fermentation was monitored by qPCR. DNA extractions were performed on pellets from the fermentation using the BioSprint 96 One-For-All kit (384) (Qiagen). DNA was also extracted from dilutions of an overnight culture of *C. perfingens* CP6 for use as a standard curve. qPCR reactions were performed using the plc gene from *C. perfringens* with previously validated forward (Cper-plc508-F CCGTTGATAGCGCAGGACA) and reverse primers (Cper-plc508-R CCCAACTATGACTCATGCTAGCA) using PCR conditions specified (Nagpal et al., 2015).

Quantitative PCR reactions were done in duplicate for each fermentation reaction. Each qPCR reaction was prepared in a 10 µl volume containing 5 µl 2× SYBR Green, 3 µl nuclease-free water, 1 µl primer mix (a mixture of forward and reverse primer of 5 µM each), and 1 µl DNA template. The reactions were cycled once at 95 °C for 5 min, followed by 50 cycles at 94 °C for 20 s, 60 °C for 20 s, and 72 °C for 50 s. Fluorescent products were measured during the last step of each cycle. Melting curves were performed on each PCR run by slowly increasing temperatures from 60 to 95 °C and measuring fluorescence at 0.2 °C s^-1^.

For each biological sample (sorghum genotype), three technical replicates were taken for fermentation. From each fermentation replicate, two qPCR measurements were conducted, giving a threshold cycle (C_t_) value that was then log_10_ transformed based on normalization curve to obtain log_10_(CFU) values. The mean of the two qPCR results was calculated. There were 20 qPCR outliers detected based on normal quantile plot and box plot of conditional residuals in JMP (JMP Statistical Discovery LLC, 2025) that were removed for subsequent analyses.

A separate subsample of 5 g of ground grain for five RILs from each extreme (*i.e.*, high and low AM activity) was re-analyzed to confirm the *in vitro* process and reproducibility of the results.

The results from the *in vitro* enzymatic digestion and qPCR analysis is henceforth defined as “*in vitro*”.

### 2.7 Genotyping

Tissue was collected from F_7_ seedlings grown from grain harvested from self-pollinated plots in 2023 and placed into 96-well plates. Tissue was desiccated by placing a modified cheesecloth mat over the top of the plate, inverting the plate, and placing it on top of a layer of silica beads contained in sealed plastic containers. After desiccation, plates were shipped to Intertek ScanBi Diagnostics (Alnarp, Sweden) for DNA extraction and genotyping. Genotyping was performed through Diversity Arrays Technology (DArT) to generate genome-wide single-nucleotide polymorphisms (SNPs), with reads aligned to the *Sorghum bicolor* v3.1 reference genome. The received.csv file report with SNP information was first filtered and formatted in R and subsequently filtered and imputed in TASSEL. Parental lines Tx2911 and P850029 had five and four sample replicates, respectively. Markers with no calls for more than two replicates for each parent or with heterozygous calls were removed (2644 removed). Additionally, SNPs with disparate calls across parental samples were removed (20 removed). The resulting SNPs (10656) were then imputed and filtered using the GUI of TASSEL 5 software (v5.2.96) (Bradbury et al., 2007). Impute by FSFHap was used for imputing missing SNPs, which is designed for biparental populations. The Window LD algorithm was used with the following parameters: 0.05 for minor allele frequency (MAF), 0.1 for maximum proportion of heterozygotes, 0.8 for maximum missing sites, and the remaining parameters left as default. Imputation was performed independently for each chromosome. Filtering resulted in 2,486 SNPs across all chromosomes and a total of 186 RILs. Results were transformed into ABH format and saved as a.csv file for genomic analysis and mapping in R. Individual SNPs were named with chromosome number and physical position in base pairs (bp), separated by an underscore (*e.g.*, 01_1234567).

### 2.8 Statistical analysis

The *lmer* function within the *lme4* package in R (Bates et al., 2015) was implemented to fit mixed-effects models for each trait (see model Equations 1–4 below). Best Linear Unbiased Estimators (BLUEs) were calculated for each trait using the *fixef* function of *lme4* package, with genotype set as a fixed effect. The R package *emmeans* was used to calculate estimated marginal means (EMM) and confidence intervals (CI) for the AM trait (*i.e.*, MIC and *in vitro*) and was later used to classify RILs into three AM categories, namely Low AM, Medium AM and High AM activity. Repeatability for the AM traits was reported as the pairwise correlation between field replicates. The *corr.test* function from the *psych* package in R (Revelle and Condon, 2019) was used to calculate Pearson correlations between traits, where BLUEs were used as phenotype values for each genotype, with *p*-value adjusted for false discovery rate (FDR). The *PostHocTest* function from the *DescTools* package (Signorell, 2025) and the *LSD.test* function from the *agricolae* R package (De Mendiburu Delgado, 2009) were used to calculate significant differences among AM activity groups and pericarp color classes. Principal component analysis (PCA), distance matrix and hierarchical clustering were performed with the functions *prcomp*, *dist*, and *hclust*, respectively, of the built-in R package *stats* (R Core Team, 2025). For the clustered heatmap, the *pheatmap* function from *pheatmap* R package was used (Kolde, 2025). The package *ggplot2* was used for displaying figures (Wickham, 2016).

For the MIC assay:

(1) $${\text{Y}}_{\text{ijk}}=\text{μ}+{\text{G}}_{\text{i}}+{\text{R}}_{\text{j}}+{\text{GR}}_{\text{ij}}+{\text{B}}_{\text{k}}+{\text{ ϵ}}_{\text{ijk}}$$

where ${\text{Y}}_{\text{ijk}}$ is the observation of the *i*^th^ genotype of the *j*^th^ replicate in the *k*^th^ batch, *μ* is the grand mean, G_*i*_ is the effect for the *i*^th^ genotype, R_j_ is the effect for the *j*^th^ replicate, ${\text{GR}}_{\text{ij}}$ is the *ij*^th^ genotype-by-replicate interaction effect, B_k_ is the effect of the *k*^th^ batch, and ${\text{ϵ}}_{\text{ijk}}$ is the error effect corresponding to ${\text{Y}}_{\text{ijk}}$. All terms are random effects for calculating variances, and genotype is set as a fixed effect for calculating BLUEs.

For the *in vitro* assay:

(2) $${\text{Y}}_{\text{iqjm}}=\text{μ}+{\text{G}}_{\text{i}}+{\text{Iblk}}_{\text{q}}({\text{R}}_{\text{j}})+{\text{GR}}_{\text{ij}}+{\text{ F}}_{\text{m}}({\text{R}}_{\text{j}}){\text{ ϵ}}_{\text{ijm }}$$

where ${\text{Iblk}}_{\text{q}}({\text{R}}_{\text{j}})$ is the random effect of the *q*^th^ incomplete block within the *j*^th^ replication and ${\text{F}}_{\text{m}}({\text{R}}_{\text{j}})$ is the random effect of the *m*^th^ fermentation replicate within the *j*^th^ field replicate.

For NIRS traits and stand count:

(3) $${\text{Y}}_{\text{ijq}}=\text{μ}+{\text{G}}_{\text{i}}+{\text{R}}_{\text{j}}+{\text{Iblk}}_{\text{q}}({\text{R}}_{\text{j}})+{\text{ ϵ}}_{\text{ijq}}$$

Given that the plant stand was inconsistent across plots due to poor seed emergence, agronomic and yield-related traits were affected and needed to be adjusted. For panicle per plot, anthracnose, yield, TGW, DTA, plant height and SGM, stand count was added as a covariate (fixed effect) to equation (3) based on pairwise correlation and comparison of conditional residuals of models with and without covariate.

(4) $${\text{Y}}_{\text{ijk}}=\text{μ}+\text{Z}+{\text{G}}_{\text{i}}+{\text{R}}_{\text{j}}+{\text{Iblk}}_{\text{q}}({\text{R}}_{\text{j}})+{\text{ ϵ}}_{\text{ijq}}$$

where Z is the fixed effect of the stand count covariate.

For the MIC model, Iblk was excluded due to lack of variability contribution. The AM trait was unaffected by stand count (**Supplementary Figure 1**).

### 2.9 QTL mapping

The genotype file was read into R using the *read.cross* function from the package *qtl* (Broman and Sen, 2009), using “riself” as the cross type and quality checks were performed. Individuals with more than 20% missing data were removed. For markers, segregation distortion was evaluated and markers with *p*-value < 1e^-20^ were removed. Additionally, recombination fractions were calculated using the *est.rf* function and marker grouping was evaluated per chromosome using the *formLinkageGroups* function (max.rf = 0.25, min.lod = 6); single-marker groups were removed prior to map construction. A total of 182 RILs and 2,448 markers were obtained. The genetic map was estimated with the function *est.map* with an error rate of 1e^-4^, map function “kosambi”, and tolerance of 1e^-4^. The total length of the genetic map was 1192.7 cM, with an overall average spacing of 0.5 cM.

LOD scores were calculated for traits of interest using the *scanone* function from the same R package (*qtl*), with the EM algorithm and 1,000 permutations, and LOD thresholds were extracted using a significance level of 0.05. The LOD thresholds for the AM traits were 3.02 for the *in vitro* and 2.97 for the MIC. The functions *calc.genoprob* and *scantwo* were used to calculate two-QTL models, using the EM algorithm and 100 permutations. For fitting multiple-QTL models and getting the estimated percent variances explained (PVE), the functions *makeqtl* and *fitqtl* were used with the Haley-Knott regression according to the model on Equation 5:

(5) $$\text{y}=\sum_{i=1}^{t}X_{i}{\text{β}}_{\text{i}}+\text{ϵ}$$

where *y* is the phenotypic vector of the quantitative trait, *t* is the number of QTL, β is the vector of regression coefficients, X is the matrix for the main effects including QTLs and covariates (*e.g.*, pericarp color), and ε are the residuals.

Based on the multiple-QTL model results, the most influential QTL and covariates were selected for use in a final model.

The thresholds for the two-QTL model are provided in **Supplementary Table 1**. The linkage disequilibrium (LD) *r*^2^ and *p*-values were computed using the function *LD* from the R package *genetics* (Warnes, 2002). Chi-square (χ^2^) test was performed with the function *chisq.test* from the R package *stats* to get significant differences between alleles.

## 3 Results

### 3.1 AM activity across the two distinct detection methods (MIC and *in vitro*)

The RIL population showed a range of variation in AM activity against *C. perfringens* in both detection methods. The high-tannin sorghum sample showed the lowest MIC and had one of the highest inhibitions for the *in vitro* assay. Meanwhile, the corn sample showed no inhibitory effect for *C. perfringens* in the MIC assay, but had one of the highest AM activity levels for the *in vitro* assay. The parental lines showed an intermediate AM activity in both methods, which led to many transgressive RILs with higher EMM values than both parents (73 for MIC and 127 for *in vitro*) (**Table 1****;****Supplementary Figure 2**).

**Table 1:** Table summarizing AM activity metrics for MIC and *in vitro* assays, including the mean, maximum (Max), minimum (Min), standard deviation (SD) and coefficient of variation (CV) values, and reporting the estimates for the parental lines, the tannin and corn control samples, and the repeatability for each detection method.

Method	Mean	Max	Min	SD	CV (%)	Tx2911	P850029	Tannin	Corn	Repeatability
MIC	8.39	25.74	4.21	3.45	41.14	6.61	8.46	3.225	Nd. (29.2)^*^	0.70
*In vitro*	5.10	6.34	4.33	0.36	7.02	5.14	5.23	4.47	4.63	0.40

^*^No AM activity detected (total extract concentration).

Where *in vitro* = log_10_(CFU) and MIC = mg ml^-1^.

With respect to pericarp color in the biparental population, seven RILs were segregating for R and W pericarp colors, one RIL was segregating for bR and W pericarp color, two RILs were segregating for bR and R pericarp color, 12 RILs had bR pericarp, 85 RILs had R pericarp, and 82 RILs had W pericarp. For segregating RW and bR-W RILs, grain of each color was harvested and analyzed separately. This resulted in a total of 197 unique samples: 92 R, 90 W, 13 bR, and two bR-R. Given only two RILs were segregating bR-R, they were removed from analyses involving pericarp color classification.

#### 3.1.1 MIC

The RIL population showed MIC values for *C. perfringens* ranging from 4.21 mg ml^-1^ to 25.74 mg ml^-1^, with a mean of 8.39 mg ml^-1^, and a standard deviation (SD) of 3.45 (**Table 1**). For 13 samples corresponding to seven RILs (one of which only had one replicate), no *C. perfringens* reduction was observed (*i.e.*, no AM activity). Thus, their MIC value defaulted to their original extract concentration, which ranged from 18.65 mg ml^-1^ to 25.74 mg ml^-1^. The tannin control sample had a MIC value of 3.23 mg ml^-1^, while the corn sample showed no effect on *C. perfringens* (no AM activity), with an extract concentration of 29.2 mg ml^-1^. P850029 had a MIC value close to the population mean, 8.46 mg ml^-1^, while Tx2911 value was lower, 6.61 mg ml^-1^, with no significant difference between the two parents (**Table 1**).

The tannin control, which showed the lowest value compared to the population of tannin-free RILs, served as the reference for defining high AM activity (High AM) RILs (**Table 1****;****Supplementary Figure 2**). Any RIL whose 95% CI overlapped with the tannin control’s value was classified as High AM. Using this methodology, a total of 48 RILs were classified as High AM. According to visual pericarp color designation, two High AM RILs were bR, 20 were R, and 25 were W, with one RIL (CS127) segregating for bR-R pericarp (**Supplementary Figure 3A**). Of these 48 High AM RILs, one of the bR RILs and one of the W RILs came from a bR-W segregating RIL (CS117_bR and CS117_W), demonstrating consistency in AM activity irrespective of pericarp color. Conversely, CS143_R and CS258_W samples from RILs segregating for pericarp color were High AM while their counterpart showed medium AM activity (CS143_W, CS258_R).

Low AM Activity RILs were determined using the lower CI limit (12.14) of the RIL with the highest MIC value (RIL CS228, excluding the seven RILs with no AM activity) (**Supplementary Figure 2B**), 14.94 mg ml^-1^, obtained from the EMM analysis. Ten additional RILs were classified as having low AM activity (Low AM), along with the seven RILs that had no detectable AM activity (No AM). Among the 17 total No/Low AM RILs, their pericarp colors were 11R, three W, and three bR (**Supplementary Figure 3A**). The remaining RILs and two parents (134 genotypes) were classified as having medium AM activity (Medium AM).

#### 3.1.2 *In vitro* enzymatic digestion and qPCR assay

For the *in vitro* assay, the range of Log_10_(CFU) values was from 4.33 to 6.34, with a mean of 5.10 and SD of 0.36 (**Table 1**). The control samples had values of 4.47 for the tannin sorghum and 4.63 for the corn. The parents Tx2911 and P850029 had mean values of 5.14 and 5.23, respectively, which were near the population mean and had no significant difference between them (**Table 1**). This moderate level of AM activity for both parents was consistent with the MIC assay.

RILs were again classified into AM categories (High, Medium and Low AM) using Log_10_(CFU) BLUEs 95% CI. All genotypes showed some level of *C. perfringens* inhibition, with 73 RILs classified as High AM (using the tannin control value as reference for high activity), 85 being Medium AM, and 41 RILs defined as Low AM. With respect to pericarp color, 33 High AM RILs were R, 29 were W, 10 were bR, and one was bR-R (**Supplementary Figure 3B**). In the Low AM Activity group, there are 41 RILs, 20 with R pericarp and 21 with W pericarp. The rest of the RILs and the parental lines (87 genotypes) were classified as Medium AM. Four of the 73 High AM Activity RILs came from pericarp color segregating RILs (one bRW and one RW), with both pericarp colors having the same high AM activity level (CS117_bR, CS117_W, CS67_R, and CS67_W). Four other RILs also came from pericarp color segregating RILs (RW), but only one color showed high AM activity (CS143_W, CS180_W, CS258_R CS262_R). Meanwhile, the other color showed low AM activity for two of the RILs (CS180_R and CS262_W) and medium AM Activity for the other two (CS143_R and CS258_W).

**Figure 1:**
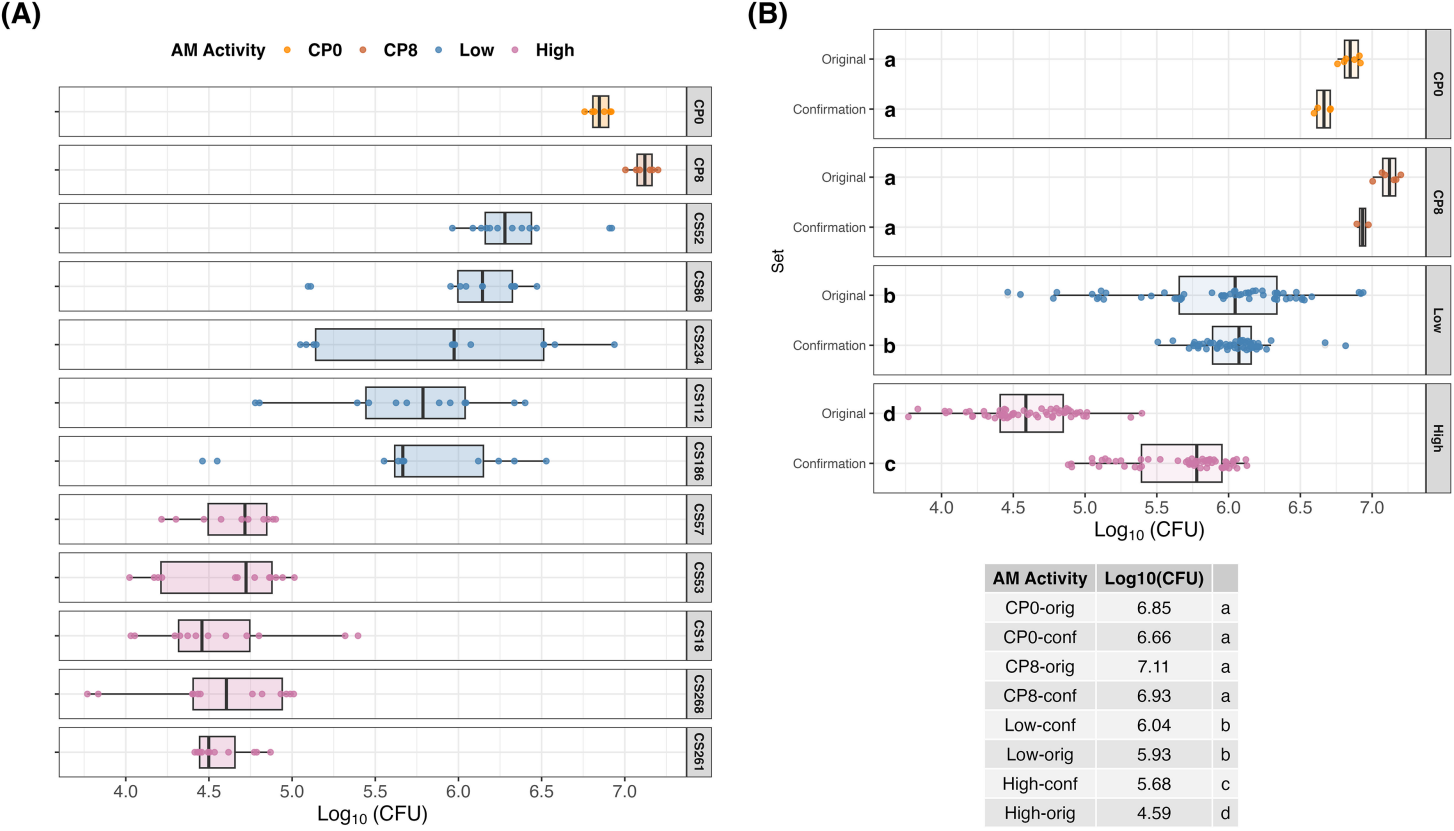
Boxplots of log_10_(CFU) of five RILs from High AM (in pink) and Low AM (in blue) groups with controls of *C. perfringens* without sorghum at 0 h (CP0) and 8 h (CP8) after fermentation demonstrate population variance and consistency of AM activity within genotype, where the boxplots in (A) are the log_10_(CFU) for the original set of samples, and in (B) are the log_10_(CFU) mean values per group (High AM, Low AM and the controls) and set (original and confirmation set) with a table summarizing the mean values and statistical differences based on the LSD test (*p*-value < 0.05). The points in each boxplot represent individual qPCR measurements, with each box containing 12 observations.

Five RILs from each extreme were selected and re-evaluated using a new subsample of ground grain to confirm the *in vitro* process and results (**Figure 1**). The High AM and Low AM groups remained statistically distinct in both runs (**Figure 1C**), and RILs showed a similar performance across runs (**Figures 1A, B**). Although the High AM RILs exhibited slightly lower AM activity in the confirmation set compared to the original run, they still demonstrated statistically higher *C. perfringens* inhibition level than the Low AM group (**Figure 1C**). Moreover, all sorghum RILs, including recombinants classified as Low AM, significantly reduced the bacterial growth relative to the no-sorghum controls at 0 h and 8 h of *C. perfringens* fermentation (CP0 and CP8, respectively) (**Figure 1**).

#### 3.1.3 Comparison between detection methods

**Figure 2:**
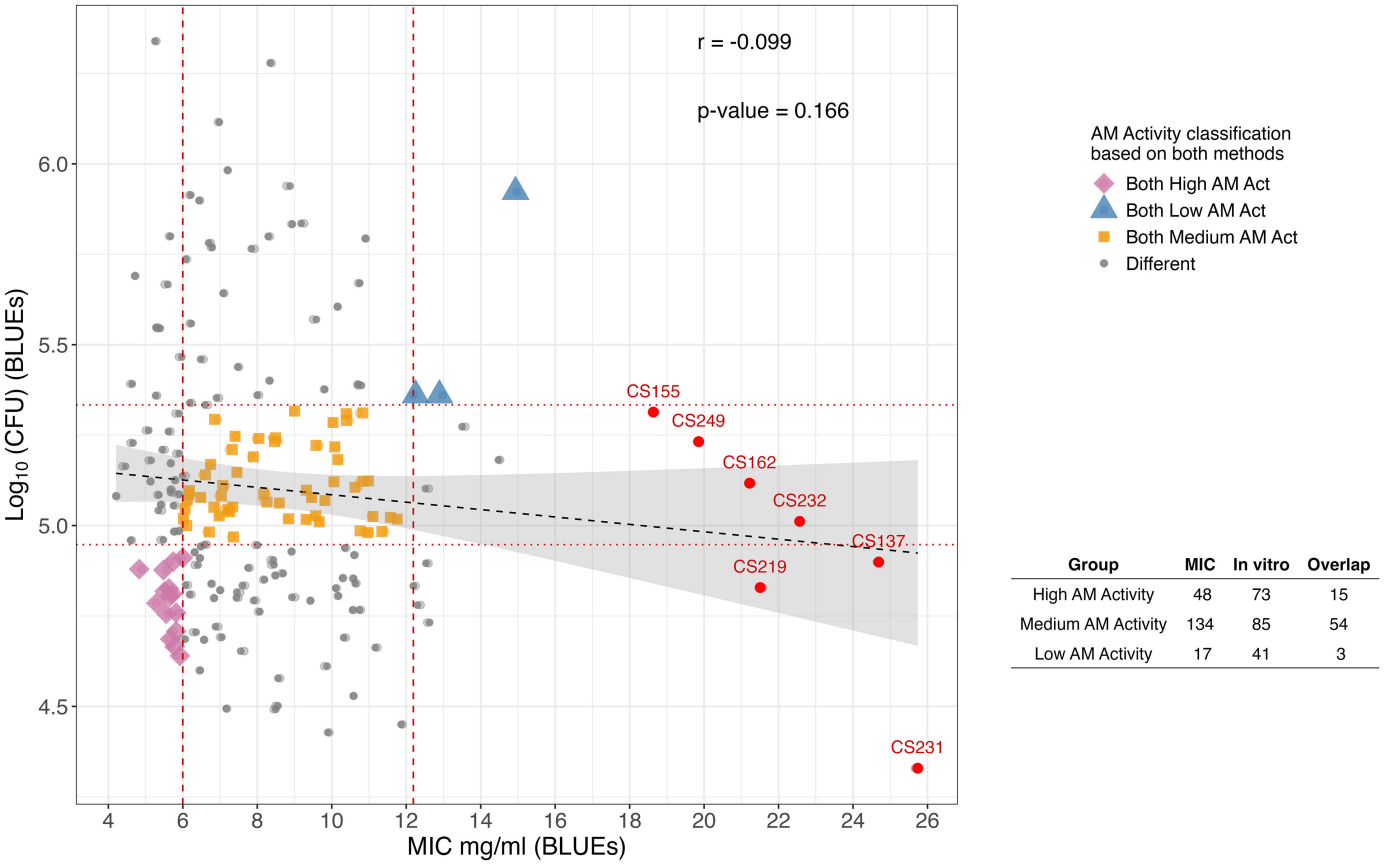
Scatterplot of Log_10_(CFU) versus MIC where each point represents an individual RIL colored according to whether they are High AM, Medium AM, Low AM or have a different AM activity classification for both detection methods. Vertical, dashed lines indicate the cutoff for high (to the left, 6.00 mg ml^-1^), medium, and low (to the right, 12.19 mg ml^-1^) MIC AM activity groups based on values obtained from the EMM analysis. Meanwhile, horizontal dotted lines indicate the cutoffs for high (to the bottom, 4.95), medium, and low (to the top, 5.33) *in vitro* AM activity groups based on values from the EMM analysis. RILs in red had no detectable AM activity in the MIC assay. The correlation coefficient *r* and *p*-value for the pairwise correlation between methods are shown. The table summarizes the number of RILs in each AM activity group, and the number of RILs that were consistently classified across detection methods.

Both methods were able to detect variation in the AM activity against *C. perfringens* within the population. However, there was no significant correlation between the AM activity values generated by the two methods (*r* = -0.099, *p*-value = 0.166) (**Figure 2**). As such, there were considerable differences in the AM classification across the MIC and *in vitro* assays. Specifically, there were 15, 54, and three genotypes that were consistently classified as High AM, Medium AM, and Low AM, respectively, with both methods, leaving 127 RILs assigned to different categories. The discovery of High AM RILs using both methods suggests the possibility of combining complementary alleles for the AM traits. The finding that 15 RILs displayed high AM activity, as determined by both MIC and *in vitro* assays, was encouraging. These progeny serve as sources for further studies and, importantly, as trait donors for germplasm enhancement.

Repeatability for the MIC results was *r* = 0.70, which was higher than *r* = 0.40 for the *in vitro* assay (**Table 1**). The majority of trait variance for the MIC-derived AM activity values was due to genotypic effects (**Supplementary Table 2**). In contrast, the greater phenotypic variance observed in the *in vitro* results was attributed to residuals, which indicated a higher technical error or greater sensitivity to environmental effects (**Supplementary Table 2**). However, the confirmation set of the extreme RILs showed good reproducibility of the test (**Figure 1**).

When looking at the effects of pericarp color on AM activity, no significant differences were found for the MIC results. However, for the *in vitro*, the bR RILs putatively harboring the intensifier allele showed higher AM activity (4.81 log_10_(CFU) with SD = 0.25) than individuals with R (5.13 log_10_(CFU), *p*-value = 0.0017, SD = 0.35) and W (5.13 log_10_(CFU), *p*-value = 0.0017, SD = 0.36) pericarp color (**Supplementary Figure 3**). A caveat is the number of RILs within each pericarp class was unbalanced, and the RILs with bR pericarp (14) were significantly less than RILs with R (92) and W (91) grain. Of the 14 bR RILs, 10 (71%) were classified as High AM from the *in vitro* assay, and the remaining four displayed medium AM activity (**Supplementary Figure 3B**). Meanwhile, the number of R and W RILs classified as High AM was almost the same (33 R and 29 W). Of the 15 RILs that exhibited high AM activity across methods, five had a W pericarp, seven had R, one RIL (CS117) had bR-W, and one RIL (CS127) segregated for bR-R. Independent analysis of bR and W grain samples from CS117 that segregated for these two pericarp colors both classified as having high AM activity.

### 3.2 Phenotypic profiling of the segregating progeny

**Figure 3:**
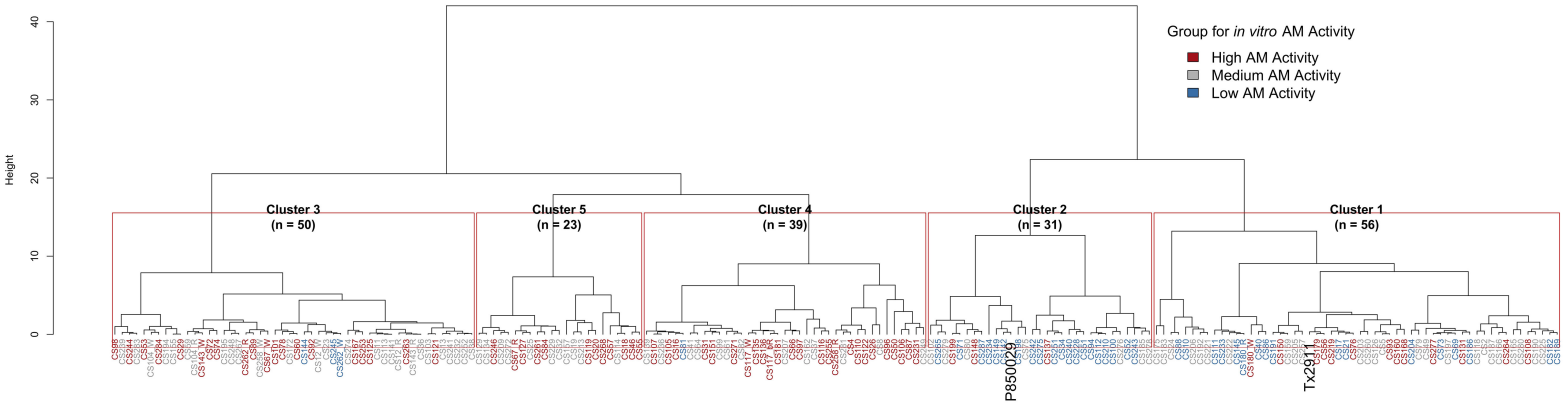
Hierarchical clustering differentiates *in vitro* AM activity groups and clusters, including the number of lines in each cluster (n), and highlighting parental lines.

Hierarchical clustering of the RIL population using all phenotypic data available resulted in five distinct clusters. The parents were positioned in two different clusters but on the same side of the tree (cluster 2 for P850029 and cluster 1 for Tx2911) (**Supplementary Figure 4A**). This is consistent with the phenotypic distribution observed for both AM traits, with the parents both having a moderate AM activity rather than contrasting phenotypes (**Supplementary Figure 2**), resulting in frequent transgressive segregants among the progenies. The AM activity groups based on MIC values failed to cluster together (**Supplementary Figure 4B**). Conversely, with the *in vitro* grouping, a trend was observed where most High AM RILs were located on one side of the tree in clusters 3, 4 and 5 (**Figure 3**). These three clusters showed significantly higher AM activity than the rest (*p*-value < 0.05), with only one Low AM RIL found in cluster 4 and three in cluster 3 (**Supplementary Figure 5**). Cluster 2 had the highest mean log_10_(CFU) (lowest AM activity), to suggest *in vitro* AM activity is associated with a distinct phenotypic pattern. Similar unbalanced clustering was observed with pericarp color, with most bR and R pericarp genotypes grouped together on one side and most W pericarp genotypes clustered on the other (**Supplementary Figure 4C**).

**Figure 4:**
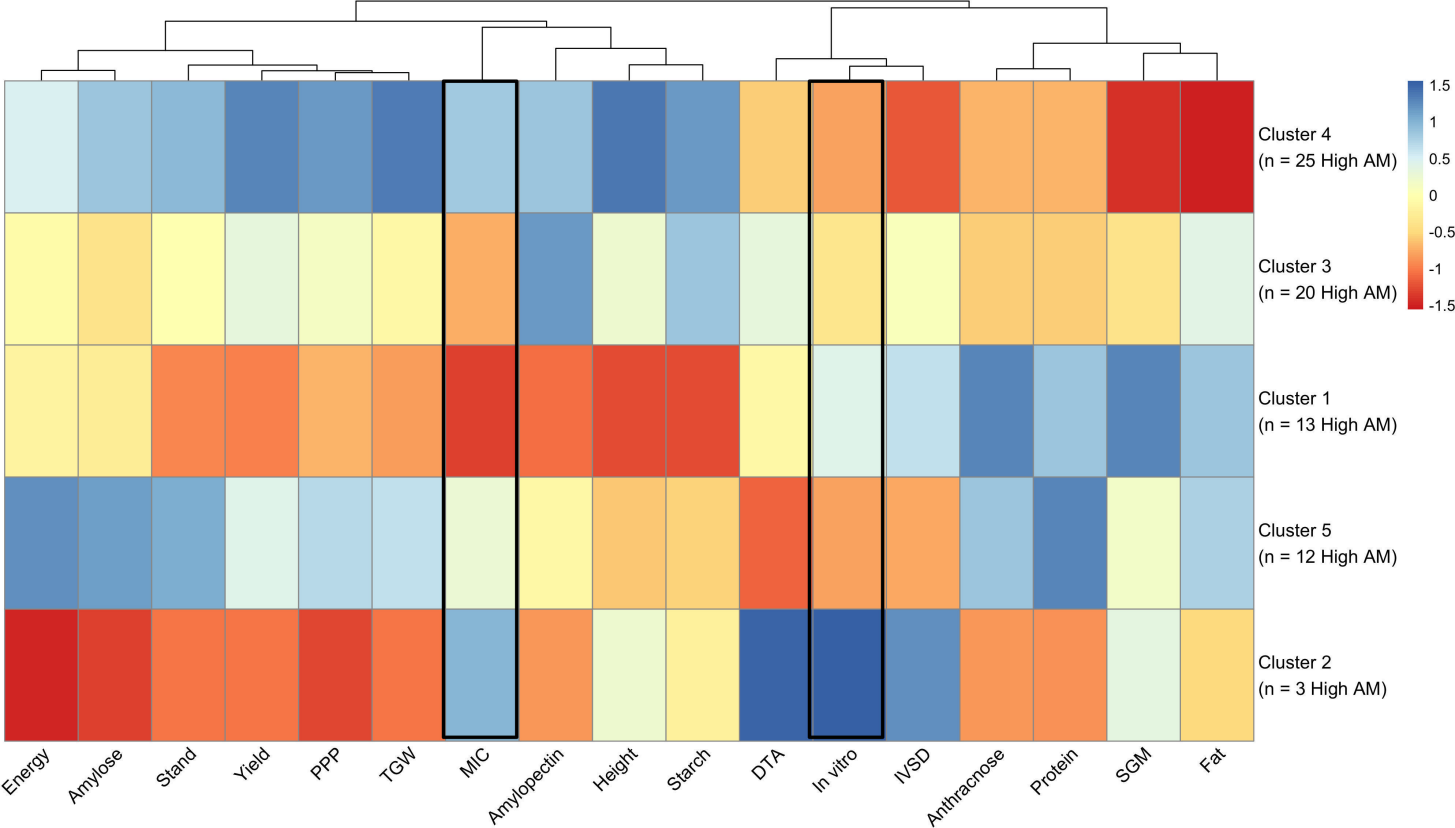
Heatmap of trait means by cluster that includes agronomic, yield and compositional traits. Rows are arranged based on the number of high *in vitro* AM activity genotypes they have.

The overall phenotypic profile of each cluster (**Figure 4**) shows that two of the top High AM clusters (clusters 4 and 5) had higher overall values for yield-related traits (stand, PPP, TGW, grain yield) and for certain compositional traits (including gross energy and amylose), and lower values for IVSD and DTA. Also, it was possible to see how the three top clusters differed in overall performance. For instance, RILs in cluster 5 had higher mean values for protein, fat, SGM and anthracnose than clusters 3 and 4, and lower values for starch and plant height. Further, clusters 3 and 5 had lower MIC values (*i.e.*, High AM); therefore, RILs within these clusters possessed both high *in vitro* and MIC AM activity. Additionally, 11 of the 15 RILs that showed high AM activity for both methods were placed in clusters 3 (four RILs), 4 (six RILs) and 5 (one RIL), with the other four RILs in clusters 1. This clustering pattern using all relevant phenotypes assisted in identifying the most promising RILs for future research studies, targeted introgression, and general population improvement.

### 3.3 Grain compositional and yield-related traits and their relationship with the AM trait

#### 3.3.1 NIRS grain composition traits

No significant correlations were found between MIC AM activity and any compositional traits (all *p*-values > 0.05) (**Supplementary Figure 6**). Conversely, several significant correlations were found between NIRS traits and *in vitro* AM activity. Specifically, starch, fat and gross energy were negatively correlated (*r* = -0.31, -0.22 and -0.57, respectively) with *in vitro* AM activity while IVSD was positively correlated (*r* = 0.49) (**Supplementary Figure 6**). Thus, when looking at the *in vitro* AM Activity groups (**Table 2**), the High AM showed higher starch and fat content, as well as higher gross energy, compared to Low AM RILs, which was influenced by a positive correlation between fat content and gross energy (*r* = 0.20, *p*-value = 0.0096). Meanwhile, Low AM RILs were estimated by NIRS to have a higher IVSD, which was positively correlated with crude fat (*r* = 0.30, *p*-value = 4.80e^-5^) and negatively correlated with starch (*r* = -0.34, *p*-value = 2.79e^-6^) and gross energy (*r* = -0.71, *p*-value = 4.36e^-30^). As starch percent increase in the grain, its digestibility decreased. Means for crude protein and amylose content were similar across AM activity classes (**Table 2**).

**Table 2:** Summary table of trait performance in the RIL population (with parental lines included) showing the mean, maximum (Max) and minimum (Min) values for each *in vitro* AM Activity group; the number of lines (n) per group; and the statistical differences determined using LSD test (*p*-value < 0.05).

Trait	AM activity	Sig	Mean	Max	Min	n
Grain Yield	High	a	1147.4	3732.2	0^*^	73
(kg ha^-1^)	Medium	b	899.8	2753.4	0^*^	85
	Low	c	449.4	2267.6	0^*^	41
TGW (g)	High	a	16.08	26.45	7.41	73
	Medium	a	15.40	27.61	8.12	85
	Low	b	12.67	23.79	7.57	41
Stand	High	a	85.8	165	4.09	73
	Medium	b	71.0	166	4.95	85
	Low	c	50.6	145	2.72	41
Anthracnose	High	b	4.35	6.93	2.52	73
	Medium	a	4.60	7.13	2.36	85
	Low	a	4.70	6.53	3.31	41
SGM	High	b	3.98	8.54	1.11	73
	Medium	a	4.61	8.46	2.02	85
	Low	a	4.87	8.04	1.92	41
DTA	High	b	71.89	83.59	64.29	73
	Medium	b	72.18	84.33	63.67	85
	Low	a	74.58	82.67	67.24	41
Height (cm)	High	a	147.67	205.32	83.49	73
	Medium	a	141.14	198.80	81.23	85
	Low	a	144.62	180.94	88.67	41
Protein	High	a	11.66	13.48	10.53	73
	Medium	a	11.71	14.24	9.93	85
	Low	a	11.84	13.18	10.32	41
Starch	High	a	66.07	69.04	61.58	73
	Medium	a	65.91	68.56	60.98	85
	Low	b	64.42	68.01	60.25	41
Fat	High	a	2.44	3.05	1.89	73
	Medium	a	2.38	3.18	1.50	85
	Low	b	2.28	2.90	1.76	41
Energy	High	a	4062.8	4156.2	4012.1	73
	Medium	b	4046.6	4120.9	3991.8	85
	Low	c	4020.1	4092.6	3938.0	41
IVSD	High	c	52.03	54.90	47.84	73
	Medium	b	52.83	55.72	47.83	85
	Low	a	54.10	55.84	50.80	41

^*^Minimum yield BLUEs were negative due to model estimates. Negative values were set to zero for biological interpretability.

Other significant associations found across grain compositional traits were protein with starch content (*r* = -0.60, *p*-value = 3.79e^-19^) and gross energy (*r* = 0.51, *p*-value = 2.81e^-13^); and amylose with starch (*r* = -0.15, *p*-value = 0.0551), fat (*r* = -0.19, *p*-value = 0.0179), gross energy (*r* = 0.25, *p*-value = 0.0011), and IVSD (*r* = -0.36, *p*-value = 9.49e^-7^) (**Supplementary Figure 6**).

#### 3.3.2 Relationships of yield component, agronomic, and disease traits with AM activity

Regarding fungal diseases that were naturally present, no significant correlations were found with either anthracnose or SGM rating and the MIC values (**Supplementary Figure 6**). For *in vitro* AM activity, no significant correlation with anthracnose rating was observed, but a positive relationship (*r* = 0.26, *p*-value = 0.0008) was found with SGM, indicating that RILs with increased resistance tended to have higher AM activity. It is relevant to highlight that a positive association between the two diseases was observed (*r* = 0.23, *p*-value = 0.0025), demonstrating a tendency to coincide. Both diseases had a significantly negative impact on grain yield in the population (anthracnose: *r* = -0.35, *p*-value = 1.22e^-6^; SGM: *r* = -0.27, *p*-value = 0.0004). In addition, anthracnose and DTA were negatively correlated (*r* = -0.34, *p*-value = 4.10e^-6^) while SGM and height had a strong negative relationship (*r* = -0.41, *p*-value = 1.15e^-8^) (**Supplementary Figure 6**).

With respect to other field traits, AM activity from the MIC assay was only significantly correlated with TGW (*r* = 0.17, *p*-value = 0.0349) (**Supplementary Figure 6**). Interestingly, *in vitro* AM activity had a negative correlation with TGW (*r* = -0.36, *p*-value = 1.05e^-6^) and also demonstrated a beneficial relationship with the other yield-related traits, PPP (*r* = -0.33, *p*-value = 7.25e^-6^), grain yield (*r* = -0.37, *p*-value = 3.16e^-7^) and stand count (*r* = -0.30, *p*-value = 6.62e^-5^). This indicates that the higher the AM activity (lower log_10_(CFU)), the higher the grain yield within the population. Further, AM activity based on the *in vitro* method was positively correlated with DTA (*r* = 0.27, *p*-value = 0.0004), but no correlation was observed with plant height. All pairwise trait correlations can be found in **Supplementary Figure 6**.

When looking at the *in vitro* AM Activity classes (**Table 2**), High AM showed higher TGW and grain yield compared to Low AM, while Low AM had a higher mean DTA and SGM rating. There was no difference in plant height observed between AM groups. Thus, AM activity did not show a negative impact on grain yield (no yield drag) in this RIL population and was instead found to positively influence grain production and increase seed size. A complex relationship between AM activity, grain composition and agronomic traits was observed in this study, with grain starch content and its digestibility appearing to have key roles on *in vitro* AM activity.

### 3.4 Loci identification for the AM trait and associated traits

#### 3.4.1 Discovery of markers linked to AM activity

**Figure 5:**
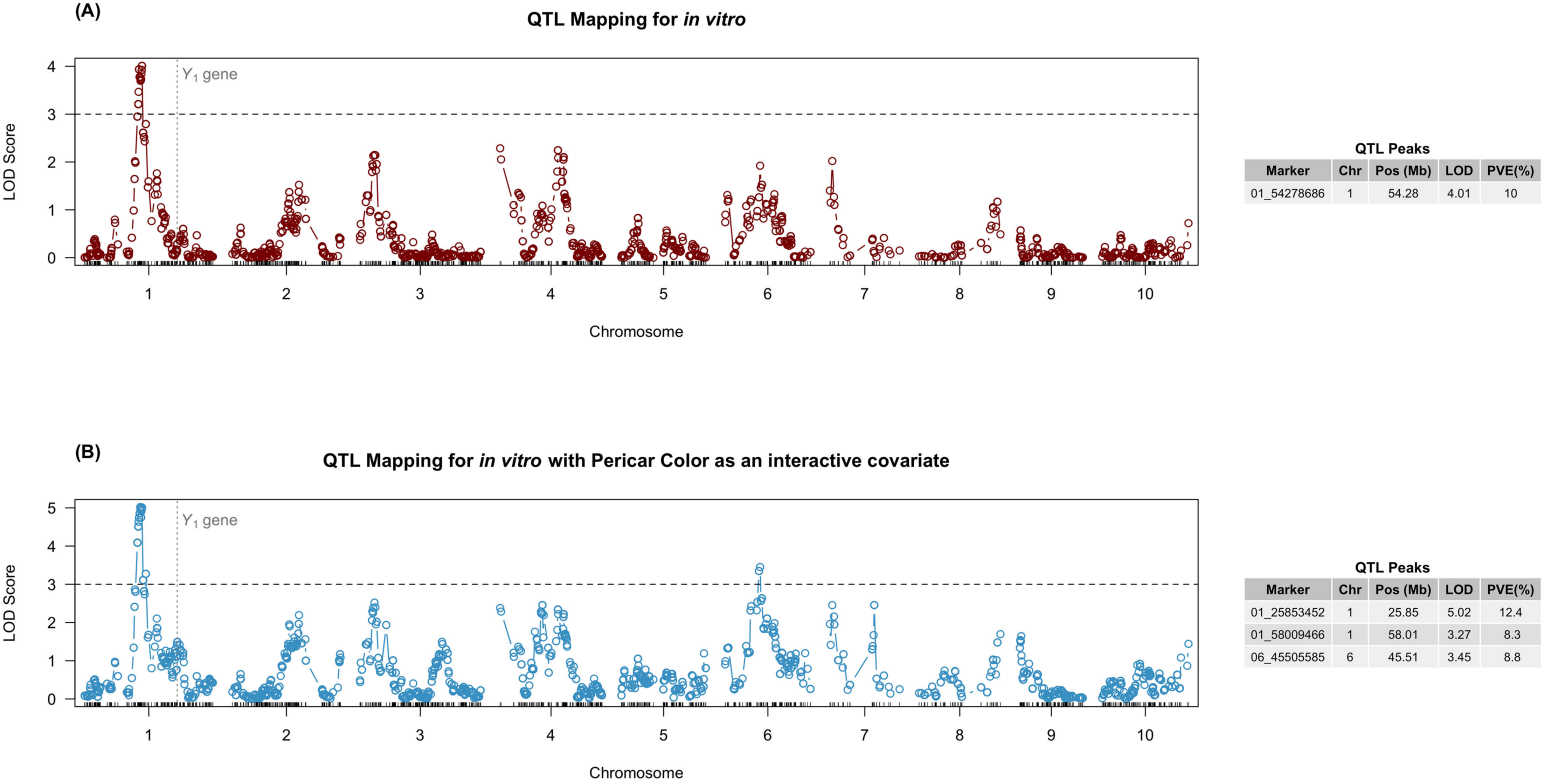
QTL mapping for **(A)***in vitro* AM activity and **(B)***in vitro* AM activity with pericarp color as an interactive covariate found a significant locus on chromosome 1. The LOD scores (y-axis) are plotted against the position in the chromosome (x-axis). The horizontal dashed lines indicate the LOD score threshold, calculated by 1,000 permutations and significance level of 0.05. Vertical dotted lines indicate the location of the *Y1* gene on chr01. The associated tables show marker names, chromosome, position (Mb), LOD scores for peaks in the single-QTL models, and the percentage of variance explained (PVE) for each marker.

No significant peaks were found for the AM activity with the MIC when running the single QTL model (**Supplementary Figure 7**), and subsequent genetic mapping for AM activity focused solely on the *in vitro* AM activity data. For *in vitro* AM activity, one significant peak was found when using the single QTL model (**Figure 5A**). The peak was on chromosome (chr) 1 at 54.28 Mb (01_54278686), with a LOD score of 4.01 and an estimated PVE of 10.0%. This peak was distant from the significant peak found for pericarp color at 68.20 Mb that overlaps with the *YELLOWSEED1* (*Y1*) gene (*Sobic.001G397900*), being in separate LD blocks (13.92 Mb away) (**Supplementary Figure 8**). Despite no overlap in QTL for AM activity and pericarp color, the phenotypic correlation between the trait (**Supplementary Figure 3**) warranted adding pericarp color as an interactive covariate to the QTL model. The QTL analysis with pericarp color as a covariate maintained the previously observed peak with an even higher LOD score (5.01), but the highest peak was at 25.85 Mb with a LOD of 5.02 (01_25853452) with a PVE of 12.4% (**Figure 5B**). These two markers were in LD with *r*^2^ = 0.88, even though they were 28.43 Mb apart (but just 2 cM, spanning the centromere) (**Supplementary Figure 8**). In addition, two new peaks were found, one on chr06 at 45.51 Mb (LOD = 3.45, PVE = 8.8%) and another one on chr01 at 58.01 Mb (LOD = 3.27, PVE = 8.3%) (**Figure 5B**). The new QTL on chr01 was located 3.73 Mb away from and in LD with 01_54278686 (*r*^2^ = 0.68) (**Supplementary Figure 8**).

#### 3.4.2 Loci interactions and models that explain the AM activity phenotypic variation

**Figure 6:**
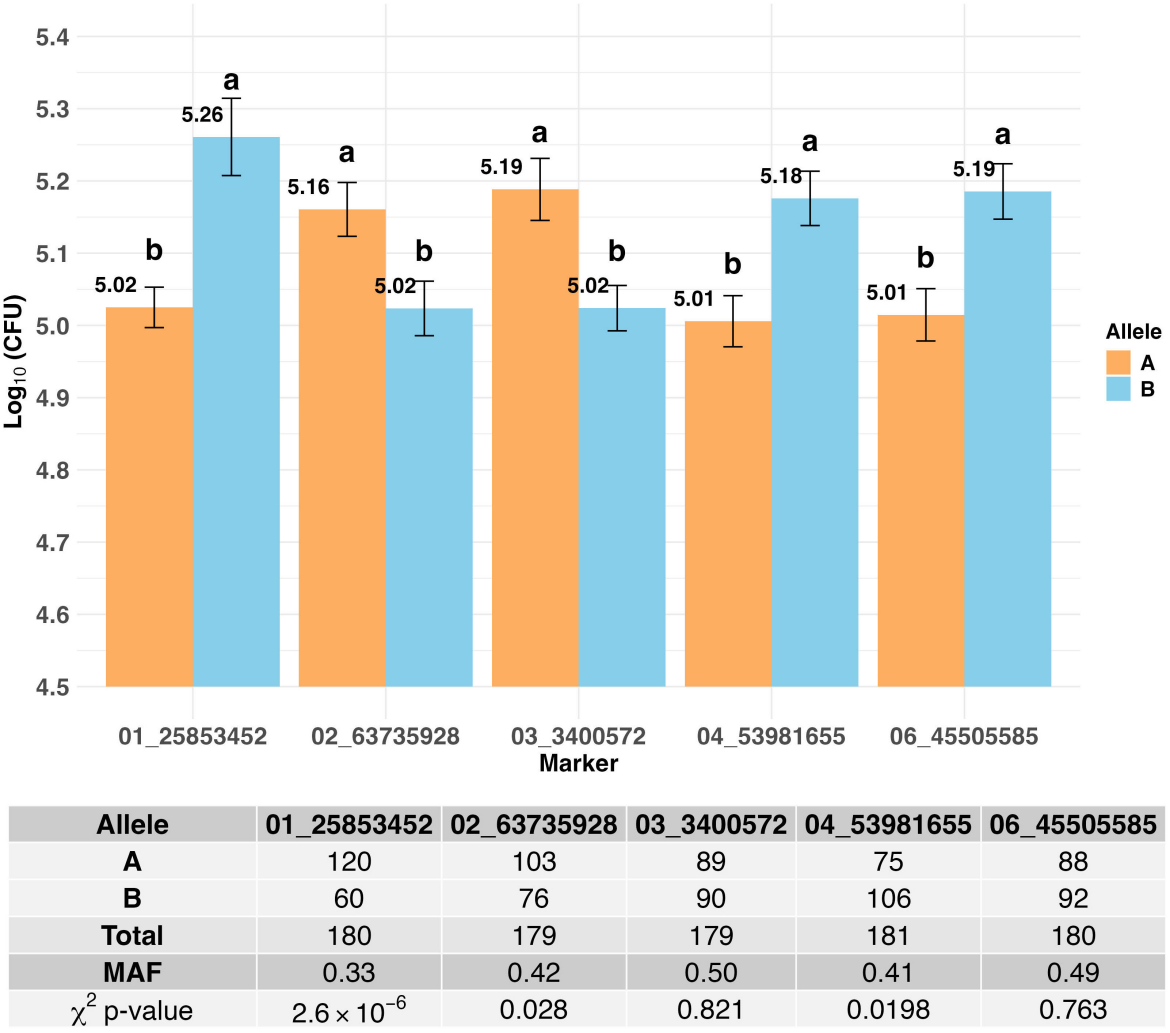
The barplot shows the *in vitro* AM activity for each allele **(A, B)** of the five markers of interest, reporting mean values and statistical differences based on the LSD test (*p*-value < 0.05). The associated table shows the number of RILs with each allele, the total number of RILs with calls for each marker, the minor allele frequency (MAF), and the results of the χ^2^ test assessing segregation distortion for each marker.

A two-QTL model was employed to assess epistatic effects between markers. In addition to the three significant markers identified with the single QTL model, ten additional other loci that approached significance showed some degree of epistatic effect (**Supplementary Table 3**). The most relevant markers in the two-QTL model were located on chr01 (23.06 – 54.28 Mb), chr02 (63.7 Mb), chr03 (3.4 Mb), chr04 (~54 Mb), and chr06 (45.51 Mb). Estimated effects for their interactions were mostly additive (**Supplementary Table 3**). Although the loci on chr01 differ in positions (at 25.62, 25.85, 25.47, 23.06, 50.80, and 54.28 Mb), they were all in LD, with *r*^2^ > 0.70 (**Supplementary Figure 8**). Thus, the peak marker from the single QTL model, 01_25853452, was used in further analysis. For the other loci, after testing different models, the markers used for further analysis were: 02_63735928, 03_3400572, 04_53981655, and 06_45505585, which showed to be most influential with highest LOD scores in the single QTL model (**Supplementary Table 4**). For these five markers, the favorable allele came from Tx2911 for three loci (chr01, chr04 and chr06) while the favorable allele at the two loci on chr02 and chr03 came from P850029 (**Figure 6**). Minor allele frequencies (MAF) were close to the expected 0.50 for two of the markers (on chr03 and chr06), while the other three loci deviated from the expected allele ratio (χ^2^*p*-value < 0.05), suggesting non-random segregation at these loci, with 01_25853452 showing the lowest MAF of 0.33, with the minor allele being unfavorable (**Figure 6**).

**Figure 7:**
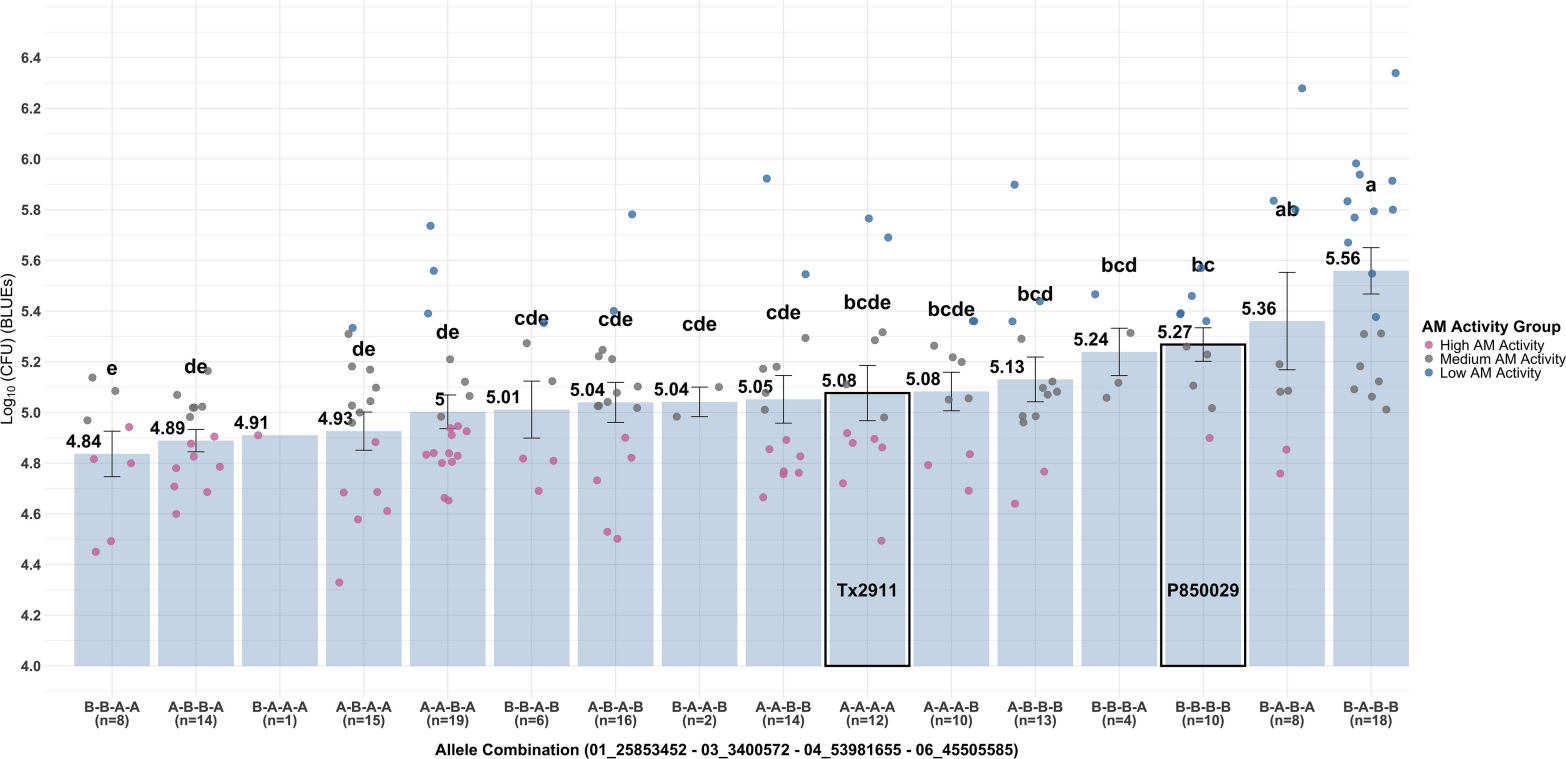
Barplot of *in vitro* AM activity by allele combination of the four-loci model of significant markers. Each bar represents an allele combination reporting the number of RILs (n), mean values and statistical differences based on the LSD test (*p*-value < 0.05). Individual RILs are represented as points, colored by AM activity group. Combinations in which all alleles come from a single parent are highlighted, with A representing Tx2911 and B representing P850029.


For each possible pair among the five markers, a single favorable allele was sufficient to increase the AM activity, which suggests a complete dominance effect (**Supplementary Figure 9**). However, when more than two loci were considered (**Supplementary Figure 10**), there were loci that displayed a greater effect on the AM activity than others, either partially or fully masking the effects at the other loci. A model using four most influential loci (chr01, chr03, chr04, and chr06, defined as Q_1_, Q_2_, Q_3_, and Q_4_, respectively) that included interaction terms (Q_1_xQ_3_) and the pericarp color covariate interaction (Q_1_xP and Q_4_xP), explained 35.3% of the phenotypic variation for AM activity, with all terms significant (**Figure 7**; **Supplementary Table 5**):

$${\text{y}}_{\text{i​}}=\text{µ}+{\text{β}}_{1}\;{\text{Q}}_{1\text{i}}+{\text{β}}_{2}\;{\text{Q}}_{2\text{i}}+{\text{β}}_{3}\;{\text{Q}}_{3\text{i}}+{\text{β}}_{4}\;{\text{Q}}_{4\text{i}}+{\text{β}}_{13}({\text{Q}}_{1\text{i}}\;{\text{Q}}_{3\text{i}})+{\text{β}}_{\text{P}}{\text{P}}_{\text{i}}+{\text{β}}_{1\text{P}}({\text{Q}}_{1\text{i}}{\text{P}}_{\text{i}})+{\text{β}}_{4\text{P}}({\text{Q}}_{4\text{i}}{\text{P}}_{\text{i}})+{\text{ϵ}}_{\text{i}}$$

where Q_ki_ is the genotype at locus *k* and individual *i*, and P_i_ is the pericarp covariate.

Many different allele combinations were observed that achieved the same high level of AM activity. None of the RILs (23) with either of the top three haplotype combinations (*i.e.*, B-B-A-A, A-B-B-A and B-A-A-A) were classified as Low AM. Interestingly, five RILs with the A-B-B-A combination (CS127, CS168, CS181, CS87, and CS89) showed high AM activity in the MIC assay. CS127 was segregated for bR-R grain color, CS89 had a W pericarp, while the other three had a R pericarp. Allele combinations of RILs with extreme AM activity can be found in **Table 3**.

**Table 3:** Table showing the haplotypes of the five most relevant markers for the five High AM and five Low AM RILs that demonstrated consistency of AM activity within genotypes, and the parental lines. Log_10_(CFU) for each biological replicate (Rep) of the RILs within each set (Original and Confirmation sets) is reported, along with the mean.

RIL	Markers					Original set			Confirmation set			AM activity
	01_25853452	02_63735928	03_3400572	04_53981655	06_45505585	Rep		Mean	Rep		Mean	
CS52	B	A	A	B	**A**	**1**	6.46	6.35	**1**	5.92	6.04	Low
						**2**	6.24		**2**	6.16		
CS86	B	**B**	A	B	B	**1**	5.85	6.02	**1**	6.11	6.13	Low
						**2**	6.20		**2**	6.15		
CS234	B	A	A	B	**A**	**1**	5.90	5.91	**1**	5.94	5.97	Low
						**2**	5.92		**2**	6.00		
CS112	B	A	A	B	B	**1**	5.87	5.70	**1**	5.92	6.09	Low
						**2**	5.53		**2**	6.25		
CS186	**A**	A	A	B	**A**	**1**	5.26	5.67	**1**	5.79	5.95	Low
						**2**	6.09		**2**	6.10		
CS57	B	**B**	**B**	**A**	B	**1**	4.69	4.65	**1**	5.98	5.95	High
						**2**	4.61		**2**	5.92		
CS53	**A**	**B**	**B**	**A**	B	**1**	4.56	4.61	**1**	5.70	5.61	High
						**2**	4.65		**2**	5.52		
CS18	**A**	**B**	A	**A**	**A**	**1**	4.40	4.57	**1**	5.18	5.46	High
						**2**	4.74		**2**	5.75		
CS268	B	**B**	**B**	**A**	**A**	**1**	4.73	4.56	**1**	5.85	5.87	High
						**2**	4.40		**2**	5.89		
CS261	**A**	**B**	**B**	**A**	**A**	**1**	4.45	4.56	**1**	5.00	5.49	High
						**2**	4.68		**2**	5.98		
P850029	B	**B**	**B**	B	B	**1**	5.16	5.25	**1**			Medium
						**2**	5.33		**2**			
Tx2911	**A**	A	A	**A**	**A**	**1**	4.77	4.93	**1**			Medium
						**2**	5.08		**2**			

For each marker, the favorable allele is bolded and highlighted in green, while the unfavorable allele is in orange. AM activity groups are highlighted in blue for Low AM activity and in pink for High AM activity.

#### 3.4.3 Markers linked to agronomic and yield-related traits

Several peaks were detected for agronomic traits across the genome (**Table 4****;****Supplementary Figure 11A**). Peaks were found for plant height on chr06 and chr07. The one on chr06 was not linked to the AM trait (LD *r*^2^ = 0.15) (**Supplementary Figures S8**, **S12E**) and was close to the *dw2* gene (*Sobic.006G067700*) (Hilley et al., 2017), at LD *r*^2^ = 0.50 with the nearest marker to the gene (06_42783012), while the locus on chr07 was ~5.1 Mb from the *dw3* gene (*Sobic.007G163800*) (Multani et al., 2003). Two peaks on chr01 were detected for DTA, both distant from the AM trait, in a different LD block (LD *r*^2^ < 0.20) but overlapped with pericarp color (*Y1*) and with the *Ma3* locus (*Sobic.001G394400*) (Childs et al., 1997) (**Table 4****;****Supplementary Figure 8**). For field diseases, three peaks were found for anthracnose, two on chr03 (at 67.38 and 72.91 Mb) (**Supplementary Figure 12C**) and one on chr04 (at 5.83 Mb) (**Supplementary Figure 12D**), none of which were linked to AM activity markers. For SGM, since an association was observed with pericarp color (with R and W pericarp being significantly different, *p*-value = 0.002), it was added as an interactive covariate to the QTL model. Three QTL were identified on chr04 at 56.87, 7.30 and 5.83 Mb (**Supplementary Figure 12D**), with the SNP at 5.83 Mb in LD with the anthracnose marker 04_5828330 (**Supplementary Figure 8**; **Table 4**). The SNP at 56.87 Mb (04_56870315) was < 3 Mb away from the AM QTL (at 53.98 Mb), with an LD between SNPs of *r*^2^ = 0.40 (**Supplementary Figure 8**). Given the range of this QTL (~2.6 Mb), the second-highest peak, at 54.80 Mb (04_54798745), was inspected, and observed higher LD *r*^2^ values of 0.67 (**Table 4**). Additionally, this QTL was distant from *Tan1*, with 04_56870315 with LD *r*^2^ = 0.16 to the closest marker to the gene (04_62307756) (**Supplementary Figure 8**). No overlap of peaks was observed between the AM trait and stand count, confirming that AM activity was not influenced by plant stand (**Supplementary Figure 13**).

**Table 4:** Table summarizing the peaks found for agronomic, yield-related and compositional traits, reporting the peak position in Megabases (Mb), the LOD score, the QTL span (Mb) with LOD > 3, and the LD *r*^2^ value of the SNPs with the closest AM activity peak.

Trait	Chr	Peak marker	Peak (Mb)	LOD	QTL (Mb)	LD *r*^2^ (AM marker)
Pericarp Color	1	01_68198125	68.20	56.55	60.40 – 76.30	
Crude Protein	1	01_5452095	5.45	4.07	3.88 – 6.05	
	1	01_3015947	3.02	4.02	2.38 – 3.02	
	2	02_15127154	15.13	3.12	14.34 – 15.13	
	3	03_7362767	7.36	6.35	7.09 – 10.64	0.22 (3.40 Mb)
	6	06_17429792	17.43	3.39		0.15 (45.51 Mb)
Starch	6	06_55472101	55.47	3.42	54.74 – 56.14	0.05 (45.51 Mb)
Crude Fat	1	01_71003685	71.00	10.37	64.37 – 75.65	
	3	03_57288873	57.29	3.81	57.22 – 57.38	
	4	04_53336840	53.34	3.05	53.34 – 53.96	0.81 (53.98 Mb)
	4	04_51710813	51.71	3.05	51.45 – 51.90	0.36 (53.98 Mb)
Gross Energy	1	01_21823590	21.82	7.01	19.68 – 58.99	0.79 (25.85 Mb)
	1	01_61226796	61.23	5.59	60.40 – 62.90	
	1	01_67564501	67.56	3.85	67.12 – 68.20	
	2	02_59561102	59.56	4.33	58.29 – 59.98	0.09 (63.74 Mb)
	2	02_15127154	15.13	4.07	9.03 – 56.75	
	2	02_61050903	61.05	3.54	60.97 – 61.45	0.25 (63.74 Mb)
	3	03_7362767	7.36	4.03	7.09 – 7.36	0.22 (3.40 Mb)
	3	03_2949712	2.95	3.53	2.72 – 3.40	0.81 (3.40 Mb)
	4	04_54798745	54.80	4.19	53.73 – 56.75	0.67 (53.98 Mb)
	4	04_63341727	63.34	3.63	62.27 – 63.56	
	4	04_58053979	58.05	3.51	57.40 – 58.05	0.24 (53.98 Mb)
IVSD	1	01_71323220	71.32	7.90	60.40 – 76.30	
	1	01_23064345	23.06	3.28	21.82 – 23.97	0.81 (25.85 Mb)
	4	04_59581632	59.58	4.92	59.13 – 63.56	0.11 (53.98 Mb)
	4	04_62413909	62.41	4.90		0.04 (53.98 Mb)
Amylose	1	01_71323220	71.32	5.25	70.81 – 72.97	
	1	01_75637914	75.64	3.52	74.79 – 75.65	
	2	02_62919493	62.92	3.28	62.79 – 62.92	0.77 (63.74 Mb)
	7	07_4240394	4.24	3.15	4.24 – 41.13	
TGW	10	10_54273394	54.27	3.36	54.23 – 54.38	
DTA	1	01_67204570	67.20	5.50	66.10 – 69.20	
	1	01_61558806	61.56	3.25		0.20 (25.85 Mb) 0.33 (58.01 Mb)
SGM +	4	04_56870315	56.87	4.42	54.80 – 57.40	0.40 (53.98 Mb)
Pericarp color	4	04_54798745^*^	54.80	4.25		0.67 (53.98 Mb)
	4	04_5828330	5.83	3.54		
	4	04_7302556	7.30	4.24	6.55 – 7.88	
Anthracnose	3	03_67384432	67.38	3.96	66.70 – 67.38	
	3	03_72909976	72.91	3.20	72.47 – 72.95	
	4	04_5828330	5.83	3.46		
Height	6	06_17429792	17.43	3.74		0.15 (45.51 Mb)
	7	07_54788927	54.79	9.72	4.24 – 59.55	
*In vitro* +	1	01_25853452	25.85	5.05	20.29 – 56.40	
Pericarp color	1	01_58009466	58.01	3.27	57.91 – 58.01	0.58 (25.85 Mb)
	6	06_45505585	45.51	3.45	45.20 – 45.51	
	2	02_63735928	63.74	2.19		
	4	04_53981655	53.98	2.45		
	3	03_3400572	3.40	2.52		

^*^Second-highest peak within the QTL.

#### 3.4.4 Loci associated with grain composition traits

Many peaks were found for grain composition, with some of them overlapping with the AM trait (**Table 4****;****Supplementary Figure 11B**). For crude protein, peaks were found on chr01, chr02, chr03 and chr06, with the loci on chr03 (7.36 Mb) < 4 Mb away from the *in vitro* with LD *r*^2^ = 0.22 (**Supplementary Figures S8**, **S12**). Starch showed a peak on chr06 at 55.47 Mb, ~10 Mb from the *in vitro* peak, in a different LD block (*r*^2^ = 0.05). Crude fat had peaks on chr01, chr03 and chr04, with the loci on chr01 (71.00 Mb) (**Supplementary Figure 12**) and chr03 (57.29 Mb) distant from the AM trait markers. Of the two peaks on chr04, the one at 53.34 Mb was in the same LD block as the *in vitro* (*r*^2^ = 0.81), while the other one (51.71 Mb) was 2.27 Mb away (LD *r*^2^ = 0.36) (**Supplementary Figures S8**, **S12**). For IVSD, peaks were found on chr01 and chr04 (**Supplementary Figures 12A, D**). The major peak on chr01 for IVSD co-located with the *Y1* gene for pericarp color, while the smaller peak at 23.06 Mb is in LD (*r*^2^ = 0.81) with the *in vitro* marker (**Supplementary Figure 8**). The peaks on chr04 were < 10 Mb away from the AM trait marker in different LD blocks (*r*^2^ < 0.11). Gross energy had several peaks on chr01, chr02, chr03, and chr04 (**Supplementary Figure 12****;****Table 4**). The major peak on chr01 (at 21.82 Mb) overlapped with the *in vitro* loci (LD *r*^2^ = 0.79) (**Supplementary Figure 8**). The other two peaks overlapped with the *Y1* locus. One peaks on chr02 (at 61.05) was at ~2.69 Mb from the *in vitro* marker, with a LD *r*^2^ = 0.25 (**Table 4****;****Supplementary Figure 8**). The locus on chr03 at 2.95 Mb was < 0.5 Mb away from the *in vitro* with LD *r*^2^ = 0.81 (**Supplementary Figure 8**). Lastly, for chr04, the most significant SNP at 54.80 Mb overlapped with the QTL for SGM and was < 1 Mb from the AM marker (LD *r*^2^ = 0.67).

## 4 Discussion

Finding alternative sources for synthetic antimicrobial treatment is essential for the sustainability of the poultry industry. Measuring the AM activity with a novel analysis of enzymatically digested sorghum grain *in vitro* (Yang et al., 2022) demonstrated that non-tannin sorghum grain is an excellent choice for a poultry feed ingredient, which can provide natural AM activity against *C. perfringens*. Even the RILs classified as Low AM showed a significant reduction of *C. perfringens* growth when compared to the bacterium fermentation alone (**Figure 1**). In this study, the AM activity was not driven by pericarp color as hypothesized, and the trait did not create a negative impact on agronomic performance. In fact, the AM trait even had positive associations with desirable traits (*e.g.*, grain yield and SGM resistance). The association with grain gross energy value and crude fat content suggests that *in vitro* AM activity may be influenced by high-energy compounds, such as lipids or lipid-derived bioactive secondary metabolites.

### 4.1 Sorghum genotypes without functional *Tan1* and *Tan2* genes maintain a sufficient level of AM activity

This study showed that sorghum grain without a pigmented testa (requires both *Tan1* and *Tan2*; Zhang et al., 2024) and condensed tannins still possess AM activity at a level to effectively reduce *C. perfringens* growth. The AM activity measured by each method (MIC and *in vitro* assay) was repeatable over biological replicates from different field plots. However, the AM effect between detection methods varied, with RILs frequently demonstrating different levels of AM activity between the MIC and *in vitro* assays (**Figure 2**). This finding was not entirely unexpected, given that each method uses a different process for sample preparation. The MIC assay was based on chemical extraction (acetone extraction for phenolic compounds) and would be limited to activity of only the acetone-soluble components. On the other hand, the *in vitro* assay had a more biological approach, using an enzymatic digestion of whole seed that more closely resembled the natural breakdown process of sorghum in the poultry gut, and would likely contain a wider variety of bioactive components. This indicates that each method made different compounds of the grain bioavailable for the interaction with the bacteria (de Morais Cardoso et al., 2017), leading to the differences observed in the AM effects within and across RILs. Nonetheless, there were 15 RILs that showed consistently high AM activity across both methods. Interestingly, three RILs (CS137, CS219 and CS231) showed no MIC AM activity and yet were classified as High AM in the *in vitro*, and CS231 even had the highest MIC value and the lowest log_10_(CFU) value (highest AM activity), suggesting that the *in vitro* digestion process is releasing compounds that were not bioavailable or accessible with only a chemical extraction, or that the AM agent is not a phenolic compound that is soluble in acetone. This could also explain why the corn sample had no detectable AM activity in the MIC assay, given the lower overall level of phenolic compounds (Dykes and Rooney, 2007; Awika, 2011), but was among the High AM activity lines in the *in vitro* assay. However, although this study found that corn exhibited high *in vitro* AM activity against *C. perfringens*, a previous study showed that it’s *in vivo* AM effect was less effective than that of sorghum (Moritz et al., 2023). From the *in vivo* study, sorghum grain was shown to activate differentially expression genes in the intestine that regulate the immune response, effectively protecting the animal from *C. perfringens* (Moritz et al., 2023).

### 4.2 Grain productivity and quality traits were correlated with AM activity, but most were advantageous

Hierarchical clustering showed that RILs with high AM activity (*in vitro* method) tended to group together to indicate phenotypic similarities (**Figure 3**). Compared to the low AM activity group, High AM RILs showed higher grain yield, TGW, and SGM resistance (**Table 2**), which suggests no yield drag or negative effect on field performance. Additionally, high AM activity was found to be associated with early maturity RILs (lower DTA), which was also correlated with higher TGW. Similarly, Shields et al. (2021) found no tradeoff between AM activity of sorghum grain and grain yield, observing no correlations between the AM trait and yield-related traits or grain composition. Regarding grain composition, the strongest pairwise correlations with *in vitro* AM activity were gross energy (negative) and IVSD (positive) (**Supplementary Figure 6**). Further, the negative correlation between starch content and IVSD but positive between IVSD and *in vitro* log_10_(CFU) tells us that starch likely plays a key role in AM activity. With higher starch content, its digestibility is reduced, which might be because it is more tightly bound and physically inaccessible (Duodu et al., 2003). Meanwhile, the positive correlation that crude fat had with gross energy and the negative correlation with *in vitro* log_10_(CFU) suggest there may be bioactive lipids (Tokusoglu and Hall, 2011) that play a role in the antimicrobial effect against *C. perfringens*.

### 4.3 The source of AM activity against *C. perfringens* is more complex than pigment-related compounds

Pericarp color is related to the phenolic profile of the grain and determined genetically, and phenolic compounds are known to be associated with health-promoting properties (*e.g.*, AM activity) (de Morais Cardoso et al., 2017; Rhodes et al., 2017; Shields et al., 2021). Thus, given that pigmented grains show higher overall phenolic content (Dykes and Rooney, 2007; Awika, 2017; de Morais Cardoso et al., 2017), it was expected that R and bR RILs would have a positive effect on the AM activity, particularly those carrying the intensifier gene (*i.e.*, bR pericarp). However, the MIC did not show a significant relationship with pericarp color, while the *in vitro* had a lower log_10_(CFU) mean value for bR RILs compared to R and W (**Supplementary Figure 3**), although the number of bR RILs (14) was significantly less than R (92) and W (91).

Furthermore, a major-effect QTL identified for AM activity on chr01 (01_25853452) was located in a different LD block than the *Y1* locus that determines pericarp color (**Supplementary Figure 8**), which suggests that grain pigment-related compounds are not the main drivers for the AM activity in this population. Therefore, the statistical interactions observed between AM loci and *Y1* locus may capture differences in metabolite profile or accumulation associated with pigment-related background.

### 4.4 Discovery of novel loci and their interactions that affect *in vitro* AM activity against *C. perfringens*

No significant markers were detected for AM activity measured by MIC, unlike Shields et al. (2021), who identified three QTL on chr02, chr04 and chr10, which were different than the *Tan 1* and *Tan2* genes. This suggests that it is a polygenic and highly quantitative trait, as indicated by the correlation between field replicates (*r* = 0.70), reflecting significant genetic effects. In contrast, three significant QTL (two on chr01 and one on chr06) were detected with the *in vitro* single QTL model (**Figure 5**), and three additional suggestive associations (on chr02, chr03 and chr04) exhibit epistatic effects influencing the AM activity in the two-QTL analysis (**Supplementary Table 3**). These loci were different from those previously reported for AM activity (Rhodes et al., 2017; Shields et al., 2021). When evaluated in a four-loci model, these regions explained 35.3% of the phenotypic variance (**Supplementary Table 5**). While the locus on chr01 at 25.85 Mb accounted for a relatively large proportion of the variation (PVE = 12.4%, **Figure 5**), the remaining loci individually explained <10%, underscoring the polygenic nature of the AM activity. Importantly, statistical interactions detected among loci do not necessarily imply direct molecule interaction between the underlying genes; rather these interactions may indicate coordinated regulation of metabolic pathways or cumulative biochemical effects that ultimately shape the AM phenotype.

Top allele combinations for AM activity showed that both parents contributed favorable alleles (**Figure 7**) that, when recombined, have a complementary gene action, achieving a higher level of AM activity. This pattern is consistent with the presence of transgressive segregants observed in the population (**Supplementary Figure 2**) and supports the polygenic nature of the trait, or at least oligogenic. In addition, with the four-loci model and the validation subset of extremes, it was observed that multiple haplotypes gave the same high AM effect (**Figure 7**, **Table 3**).

Furthermore, it was confirmed that the SNPs found at chr04 (04_53981655) and chr02 (02_63735928) were in separate LD blocks from the *Tan1* and *Tan2* loci, respectively. These markers were found to be in LD with gross energy SNPs (**Supplementary Figure 8**), which could explain the high correlation observed between the two traits. The locus on chr04 was also in LD with crude fat (*r*^2^ = 0.81) and SGM (*r*^2^ = 0.67) markers (**Supplementary Figure 8**), traits correlated with AM activity. However, because most QTL intervals in this study spanned 2–3 Mb, which is expected for a RIL population with a mid-density marker coverage, fine mapping is needed to hone in on the underlying causal genes.

## 5 Conclusion

Overall, the findings of this study demonstrate that non-tannin sorghum grain exhibits repeatable AM activity against *C. perfringens* without compromising agronomic performance or grain quality. Pinpointing the genetic mechanisms that modulate sorghum grain AM activity is essential to incorporate the trait into commercially available sorghum hybrids. Five QTLs involved in the AM trait that displayed both additive and epistatic effects were identified in this population, including one major-effect QTL (chr01 at 25.85 Mb), which can be leveraged to develop functional feed. By targeting these genes, the AM trait can be incorporated into sorghum varieties without impacting other essential traits (*e.g.*, grain yield). Fortunately, RILs from the biparental population possessed optimal haplotypes to serve as AM activity donors to introgress this trait into sorghum improvement programs. This positions non-tannin sorghum as a viable and sustainable option for poultry nutrition, offering the potential to reduce reliance on synthetic antibiotics and mitigate the risk of AM resistance.

Future research should focus on investigating the environmental factors influencing grain AM activity and exploring how genotype-by-environment interactions may impact its expression. Additionally, identifying the specific compounds or underlying metabolic pathways responsible for the AM effects will be essential for understanding how the trait functions at the molecular level, an insight that could enable more targeted breeding strategies.

## Data Availability

The datasets presented in this study can be found in online repositories. The names of the repository/repositories and accession number(s) can be found below: https://figshare.com/, 31382908.
