# Supplementary material for: Genetic dissection of sorghum [Sorghum bicolor (L.) Moench] grain antimicrobial activity against Clostridium perfringens and its relationship with grain composition and field performance
Source: Front Plant Sci. 2026 Mar 16;17:1754365. doi: 10.3389/fpls.2026.1754365 (PMC13033767; doi:10.3389/fpls.2026.1754365)
Supplement: Supplementary file 1 [file DataSheet1.pdf]

Supplementary Material

1. Supplementary Figures and Tables

1.1. Supplementary Figures

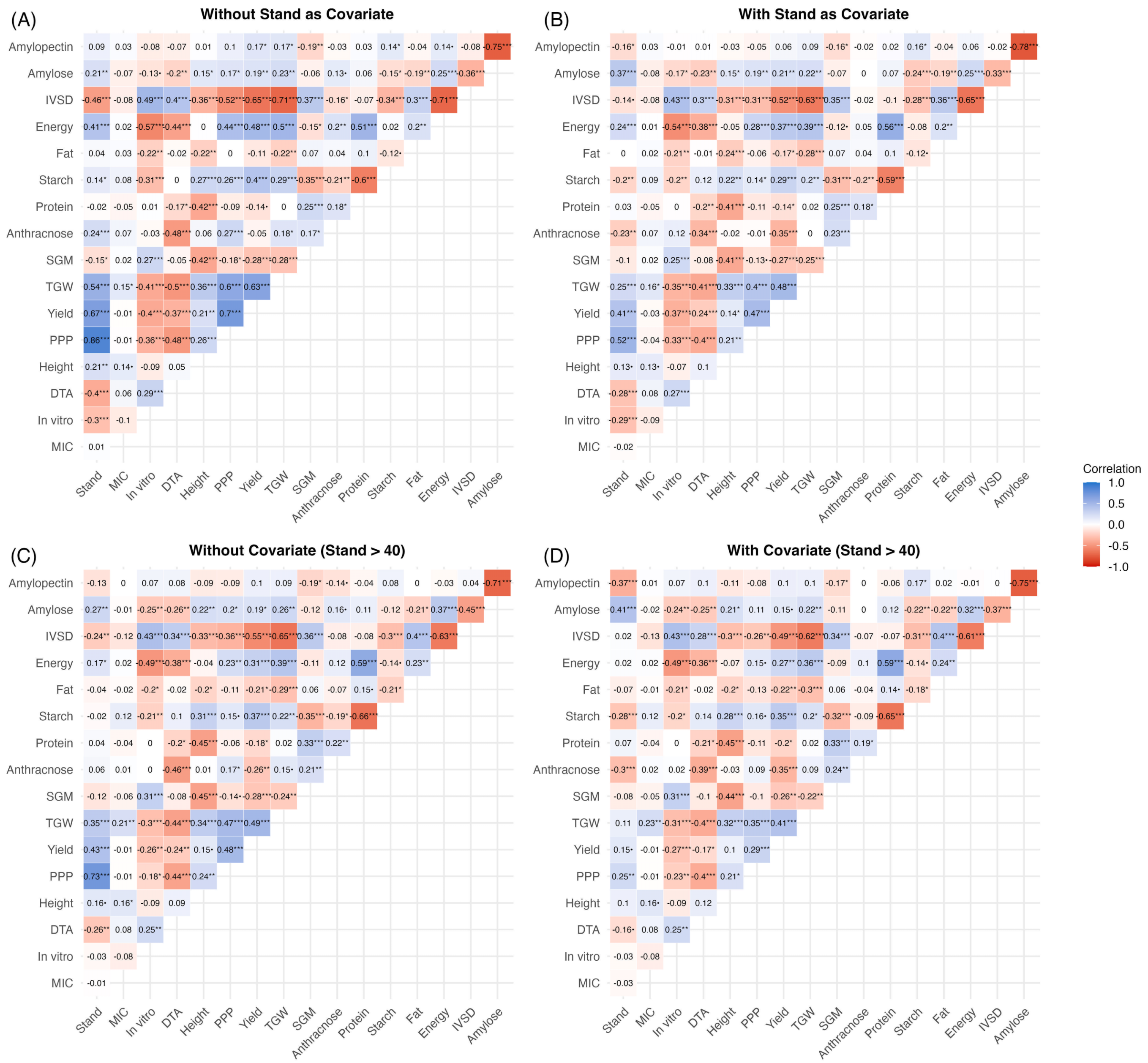

Supplementary Figure 1. Correlation matrices among all traits of interest for models evaluated with and without stand count as a covariate. Panels (A) and (B) correspond to analyses using the full dataset without and with the covariate, respectively. Panels (C) and (D) correspond to analyses restricted to genotypes with a BLUE stand count greater than 40 (removing 54 low-stand genotypes), without and with the covariate, respectively.

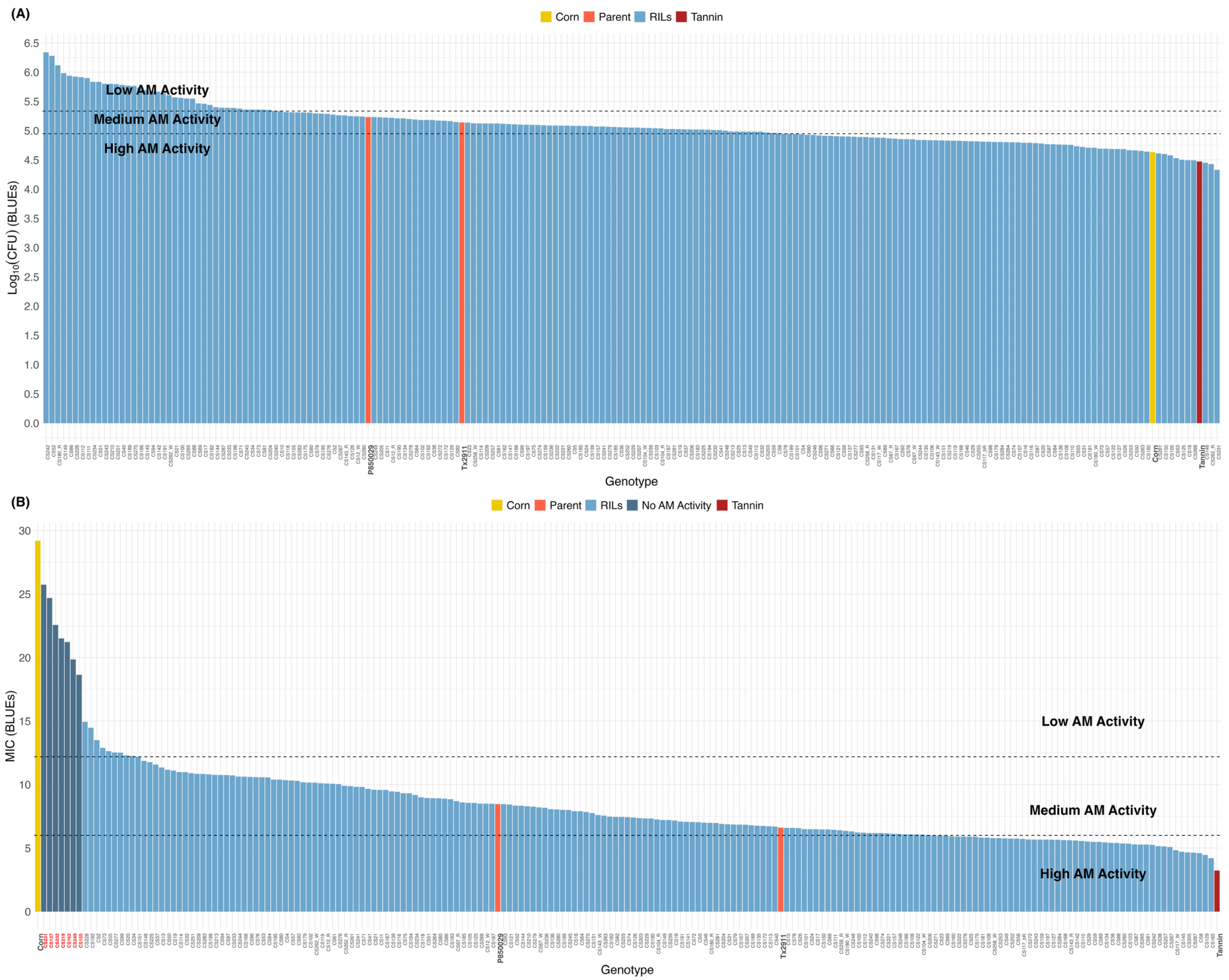

Supplementary Figure 2. Barplots showing genotypes ranked by (A) in vitro and (B) MIC AM activity, highlighting parental lines, tannin sorghum and corn sample. Horizontal lines indicate the cutoff for each AM activity group with values obtained from the EMM analysis. In (B), RILs in red with darker columns had no AM activity detected.

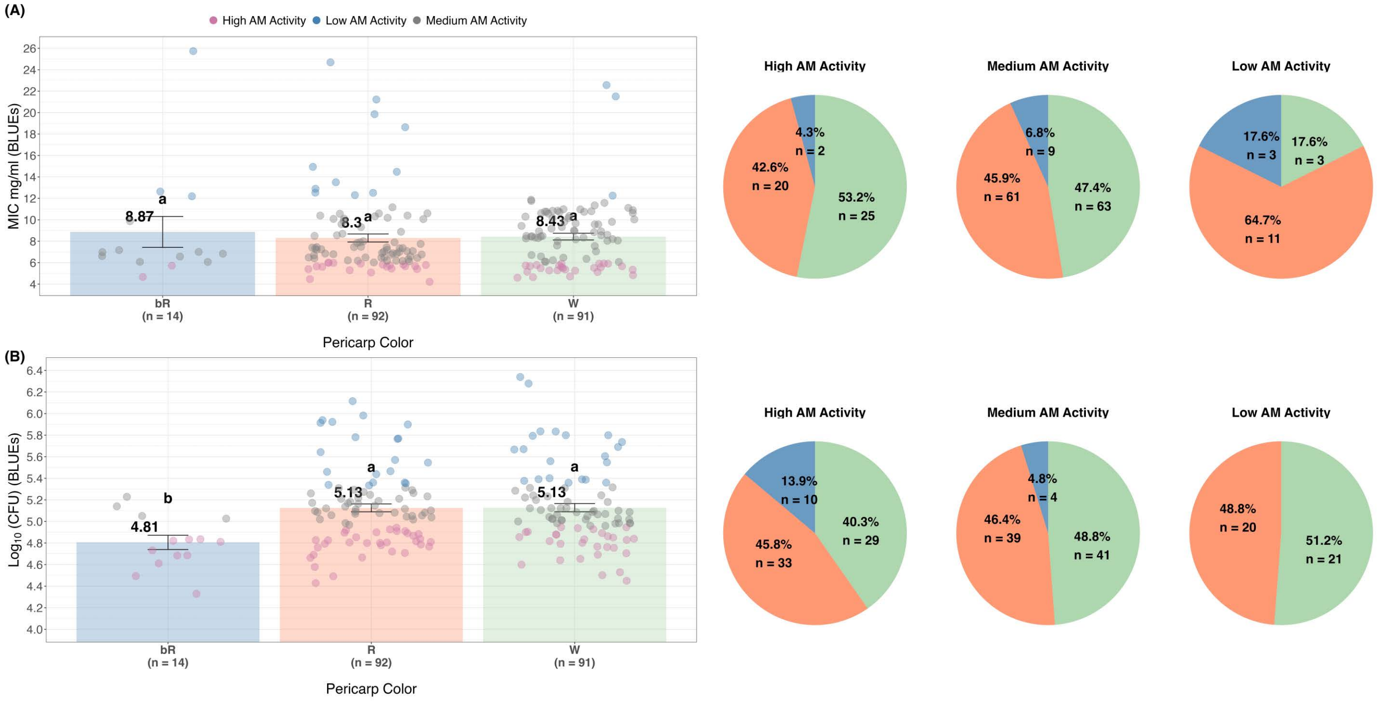

Supplementary Figure 3. Barplots and pie charts showing differences in AM activity for (A) MIC and (B) in vitro by pericarp color and AM activity group. The barplots show each pericarp color indicating the number of RILs per color, the mean value and the statistical differences determined using LSD test (*p*-value < 0.05). Each RILs is represented as a point and colored according to the AM activity group. The pie charts show the number (n) and percentage of RILs for each pericarp color within AM activity groups.

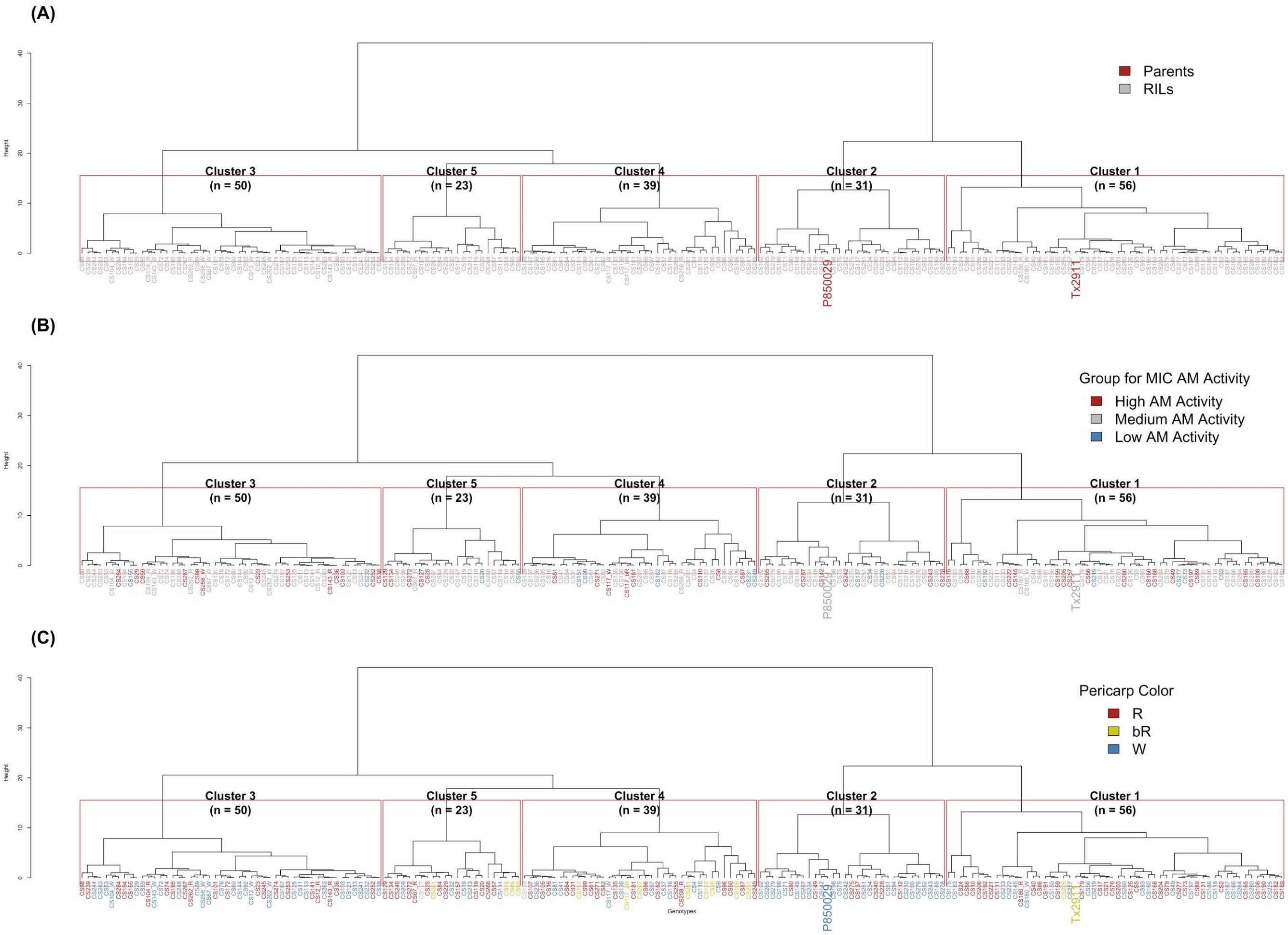

Supplementary Figure 4. Hierarchical clustering of the RIL population highlighting (A) parental lines, (B) MIC AM activity groups, and (C) pericarp color.

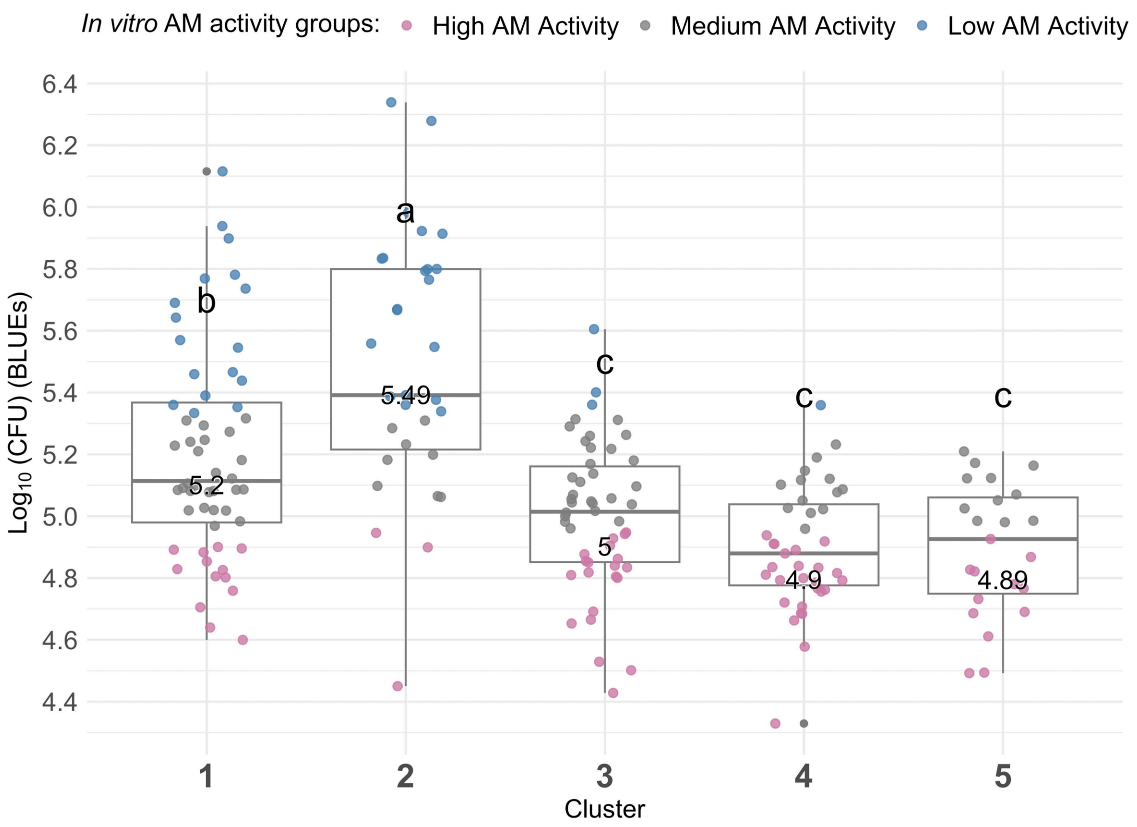

Supplementary Figure 5. Boxplot of in vitro AM activity by clusters. Each boxplot represents a cluster showing the mean value and the statistical differences determined using LSD test (p-value < 0.05). Each RILs is represented as a point and colored according to the AM activity group.

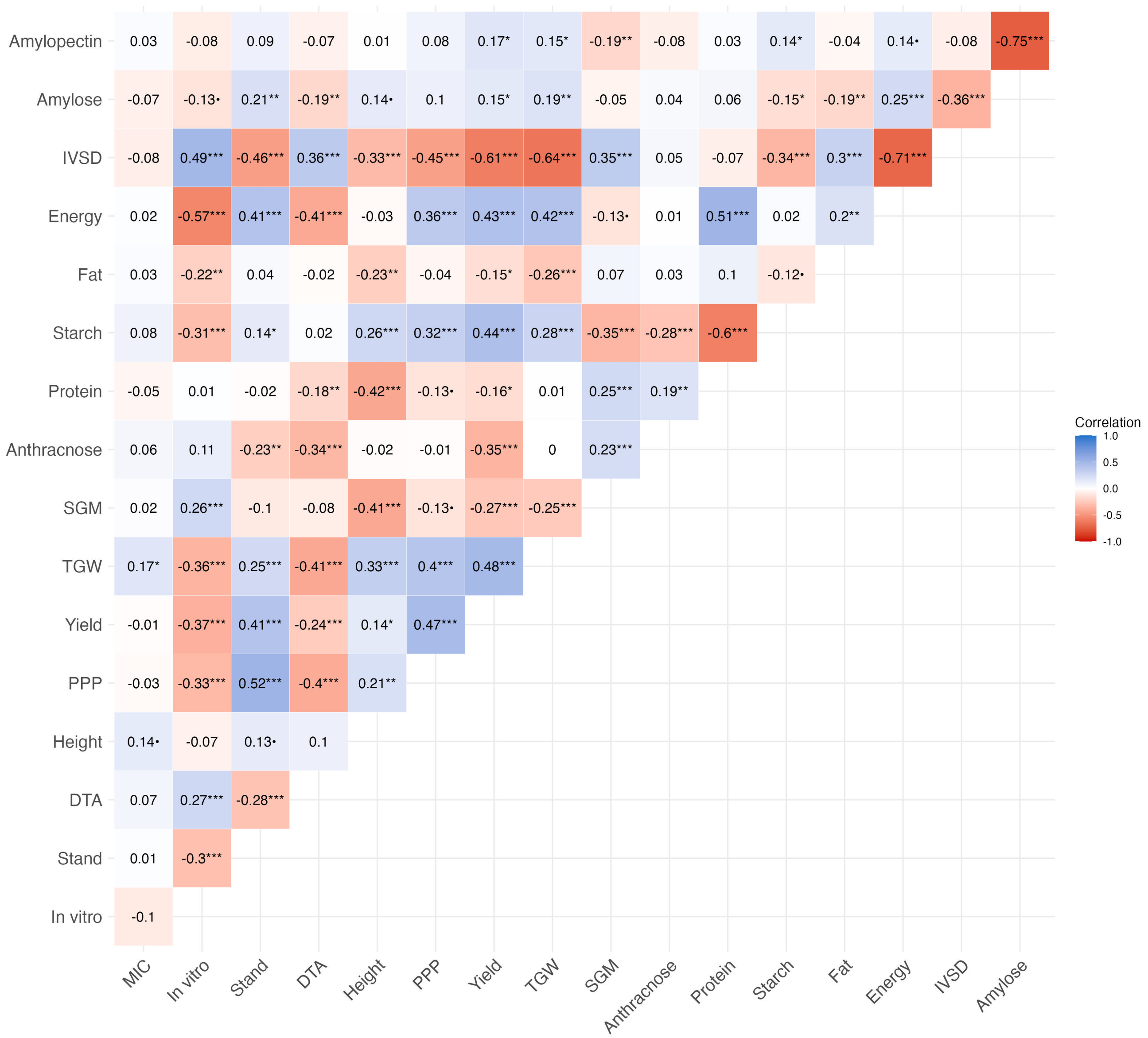

Supplementary Figure 6. Correlation matrix for agronomic, yield, compositional and AM traits with significance at probability level of "•" 0.1, "*" 0.05, "**" 0.01 and "***" 0.001, based on adjusted *p*-value for FDR.

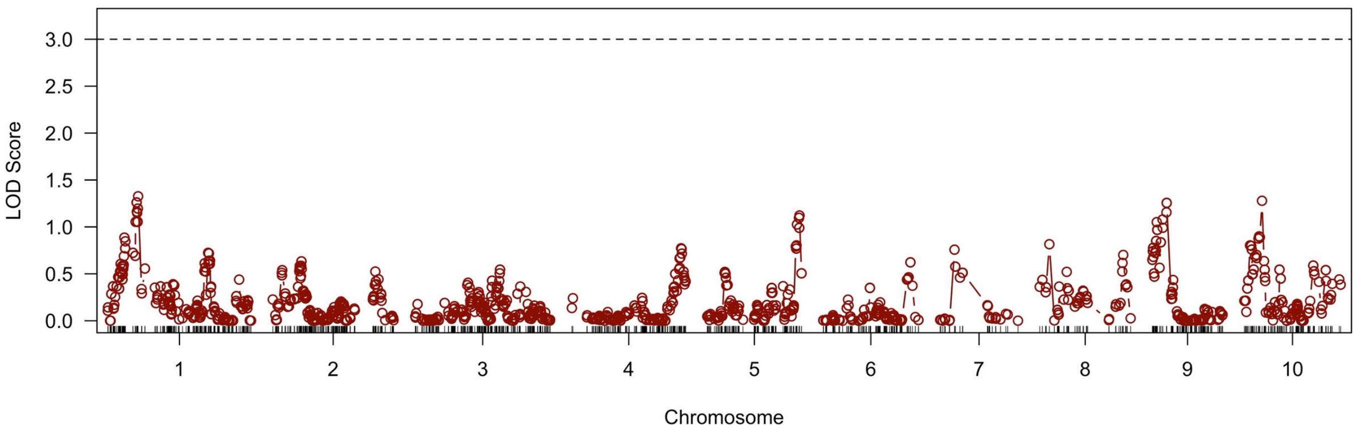

Supplementary Figure 7. QTL mapping for MIC AM activity. The LOD scores (y-axis) are plotted against the position in the chromosome (x-axis), with the dashed lines indicating the LOD score threshold calculated by 1,000 permutations and significance level of 0.05.

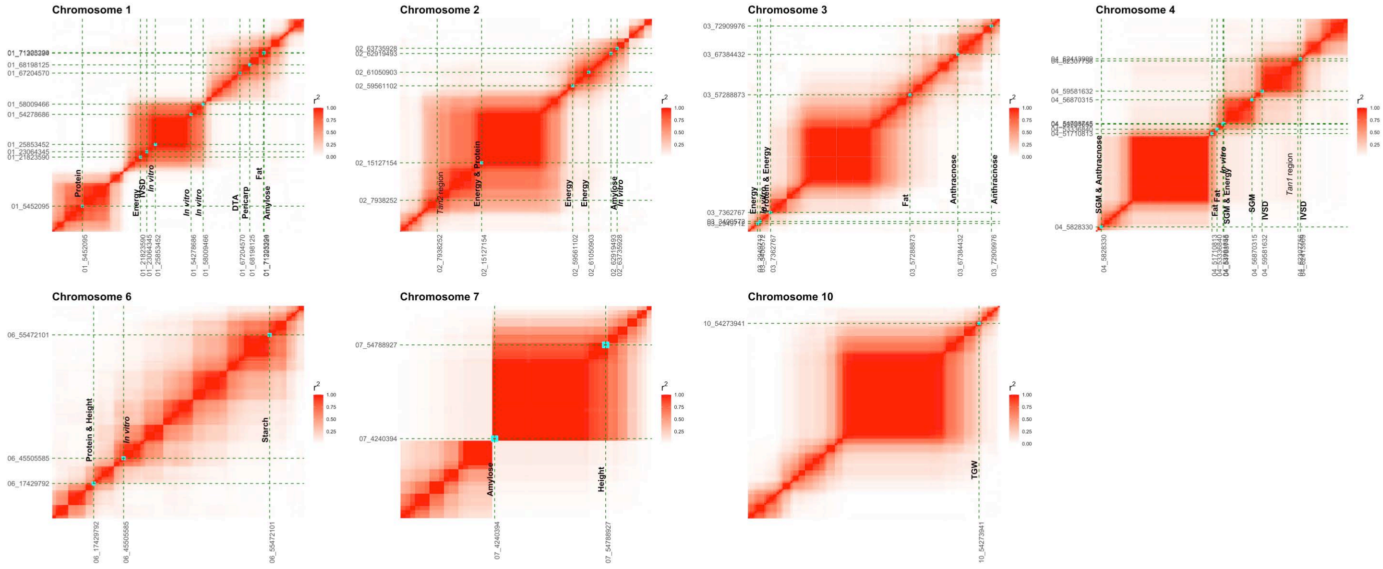

Supplementary Figure 8. Linkage disequilibrium plots for chromosomes 1-4, 6, 7 and 10, highlighting significant markers for all traits.

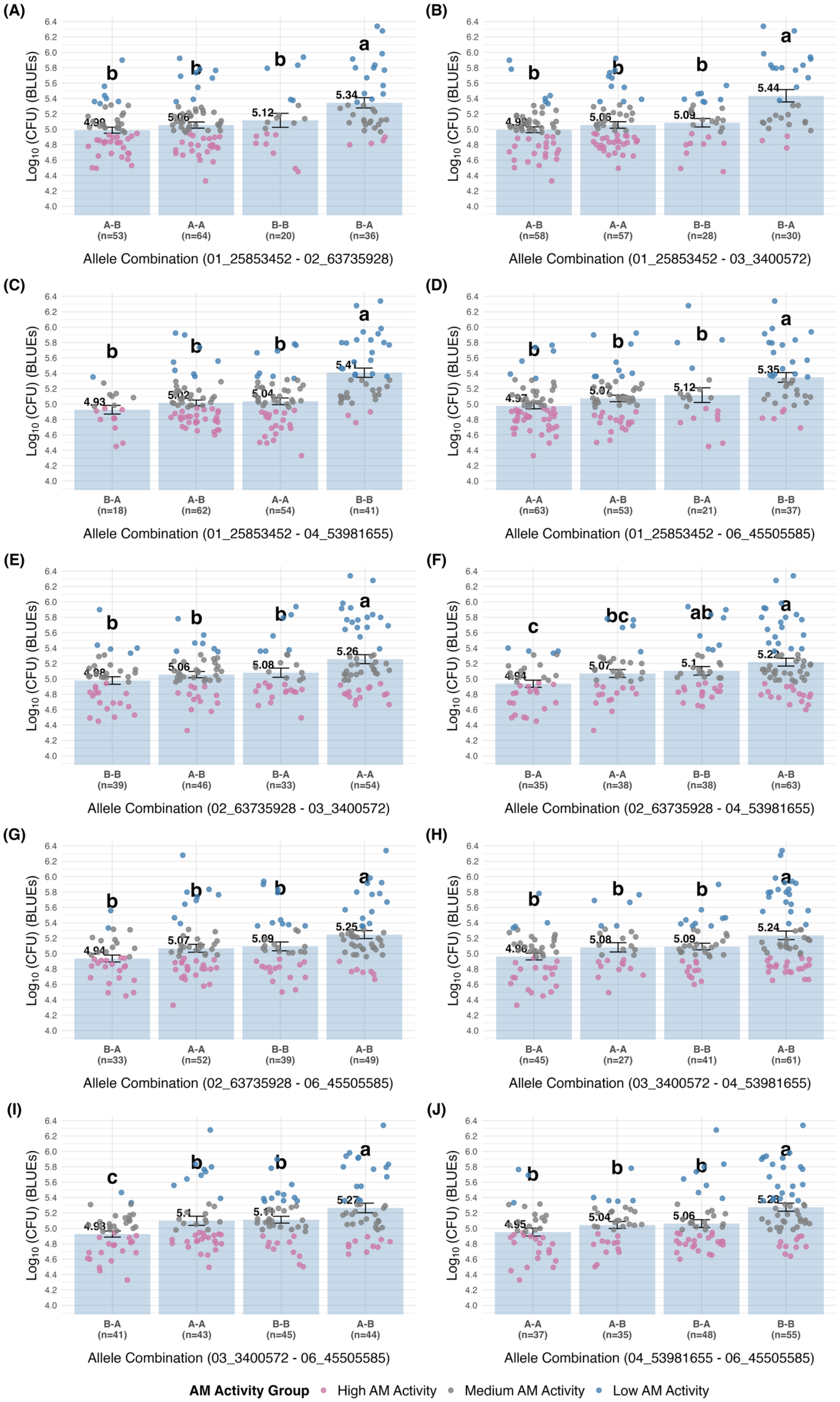

Supplementary Figure 9. Barplots of allelic effect of two-loci models for in vitro AM activity for the five markers of interest. Each bar represents an allele combination reporting the number of RILs (n), mean values and statistical differences based on the LSD test (p-value < 0.05). Individual RILs are represented as points, colored according to the AM activity group.

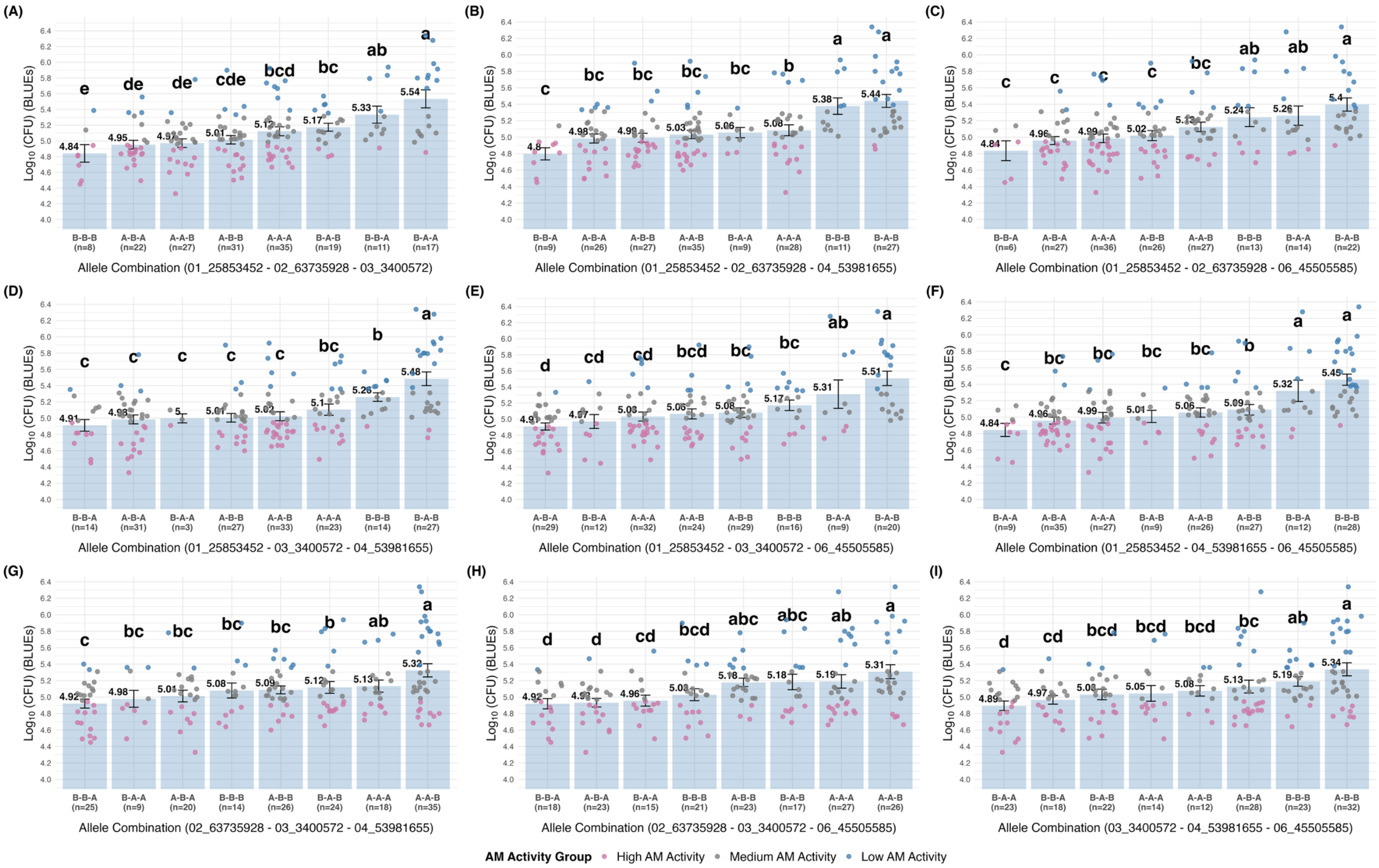

Supplementary Figure 10. Barplots of allelic effect of three-loci models for in vitro AM activity for the five markers of interest. Each bar represents an allele combination reporting the number of RILs (n), mean values and statistical differences based on the LSD test (p-value < 0.05). Individual RILs are represented as points, colored according to the AM activity group.

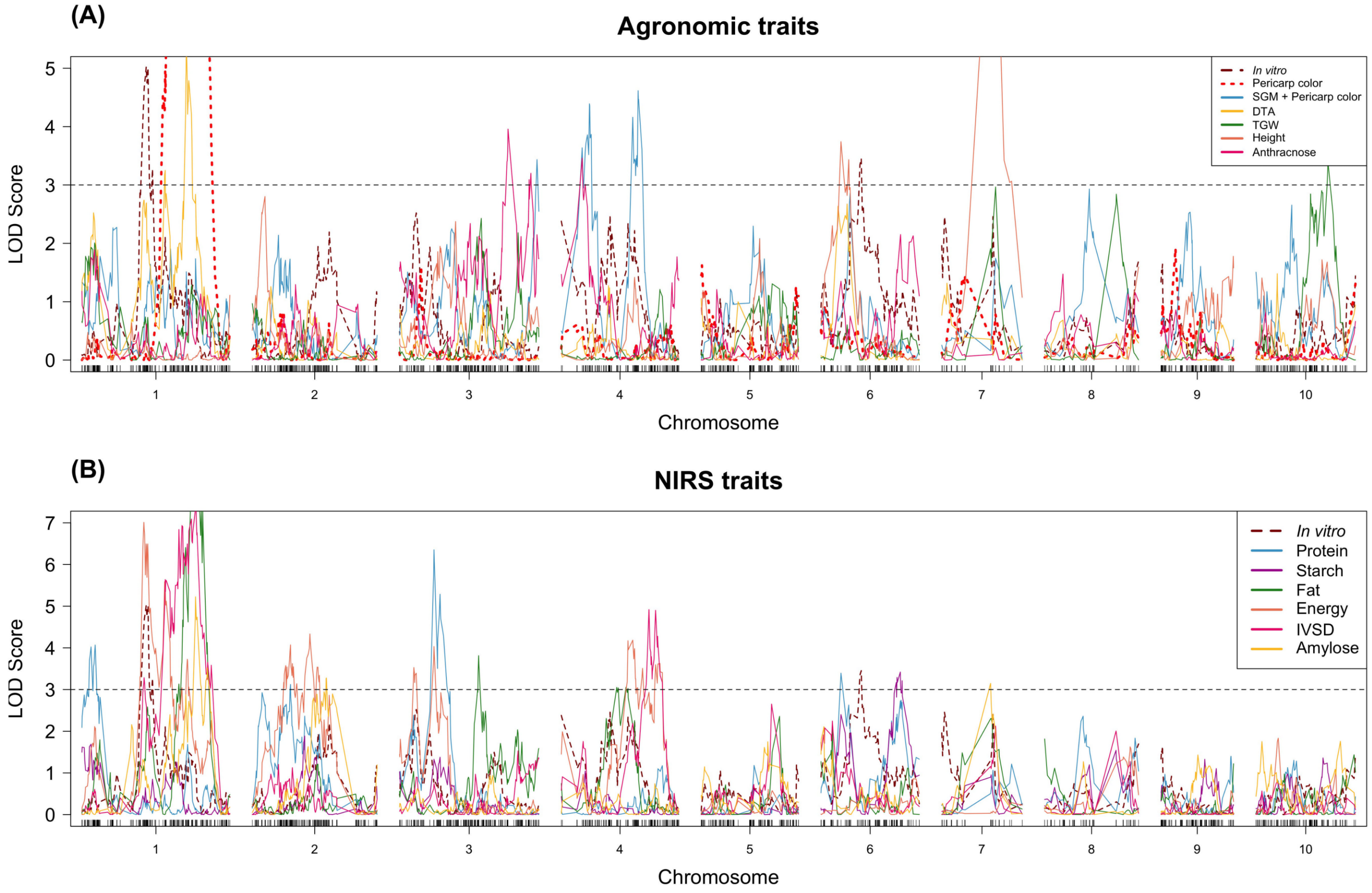

Supplementary Figure 11. Genome-wide QTL mapping for (A) agronomic and yield-related traits, and (B) NIRS traits. The LOD scores (y-axis) are plotted against the position in the chromosome (x-axis). The horizontal dashed lines indicate the LOD score threshold, calculated by 1,000 permutations and significance level of 0.05.

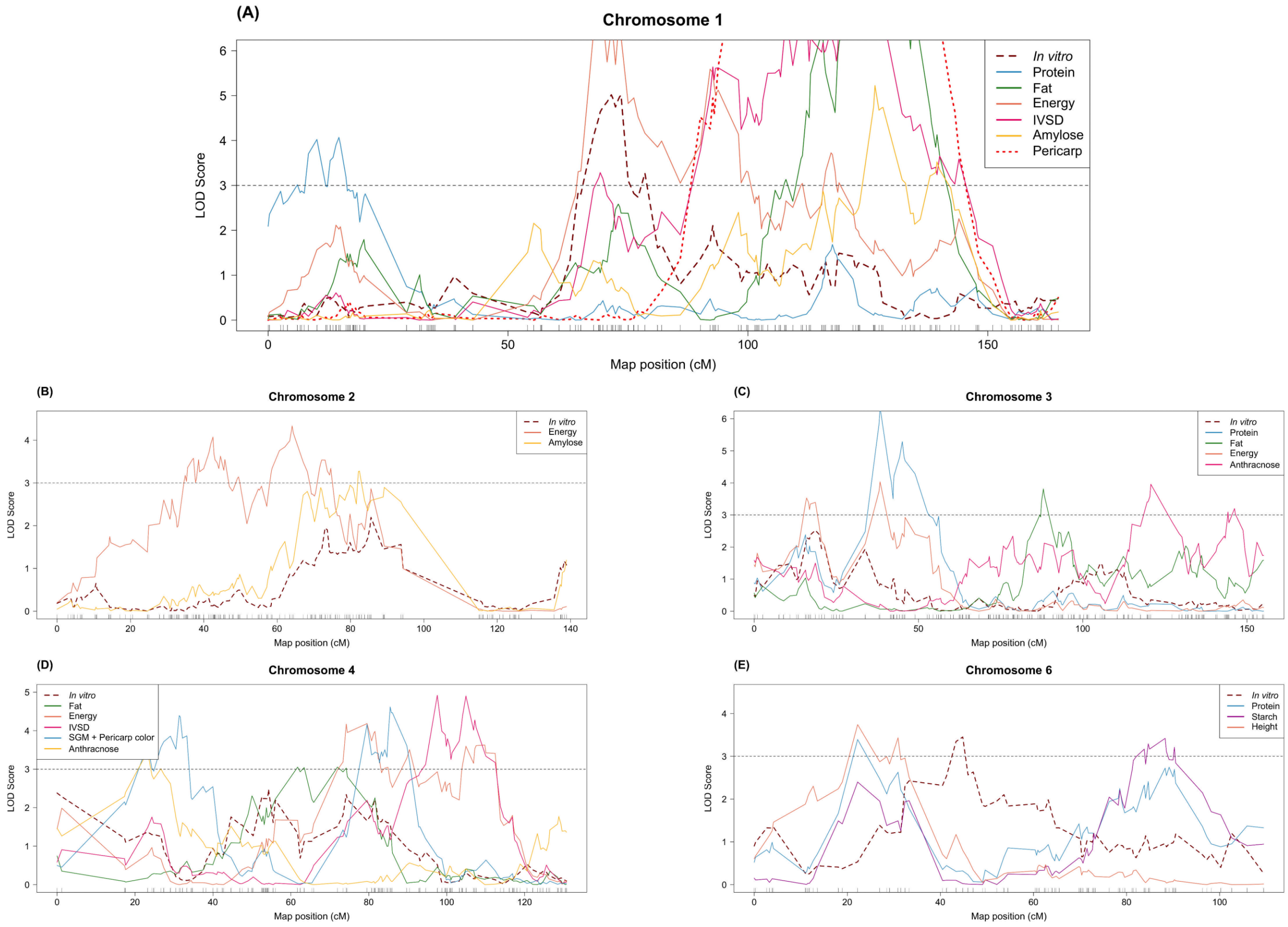

Supplementary Figure 12. QTL mapping for individual chromosomes and the significant traits in each one, with (A) being chromosome 1, (B) chromosome 2, (C) chromosome 3, (D) chromosome 4, and (E) chromosome 6. The LOD scores (y-axis) are plotted against the position in the chromosome (x-axis). The horizontal dashed lines indicate the LOD score threshold, calculated by 1,000 permutations and significance level of 0.05.

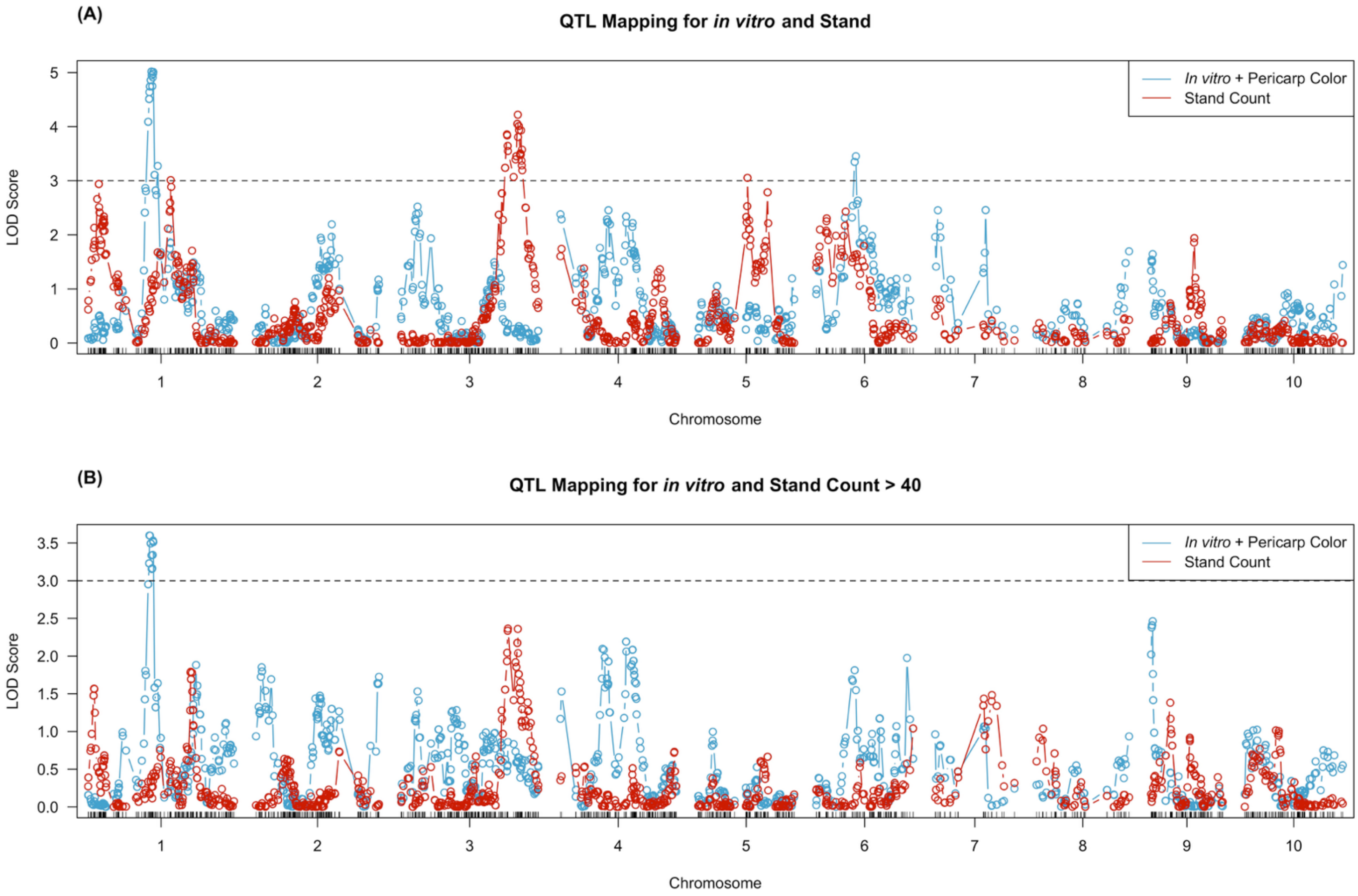

Supplementary Figure 13. Genome-wide QTL mapping for in vitro AM activity and stand count using (A) the full dataset and (B) genotypes with a BLUE stand count greater than 40 (removing 54 low-stand genotypes). The LOD scores (y-axis) are plotted against the position in the chromosome (x-axis). The horizontal dashed lines indicate the LOD score threshold, calculated by 1,000 permutations and significance level of 0.05.

1.2. Supplementary Tables

Supplementary Table 1. Table summarizing the LOD score thresholds for two-QTL analysis models using the scantwo function in R.

Trait	alpha	LOD_full+	LOD_fv1§	LOD_int¶	LOD_add†	LOD_av1¥
In vitro	5%	7.09	5.63	4.41	5.44	3.84
	10%	6.75	4.95	3.99	5.24	3.37

+LOD score for the full model (additive + interaction)
§LOD score for comparing full model vs. model with only the first locus
¶LOD score for the interaction effect alone (full model vs additive-only model)
†LOD score for the additive model
¥LOD score for comparing additive model vs. model with only the first locus (without interaction)

Supplementary Table 2. Table summarizing the variance components for each model of the AM traits.

MIC		*In vitro*	
**Component**	**Variance**	**Component**	**Variance**
**G**	9.838	**G**	0.080
**R**	0.143	**Iblk(R)**	0.035
**G*R**	1.107	**G*R**	7.7e-10
**Batch**	0.490	**F(R)**	0.054
**Residual**	2.429	**Residual**	0.273

Supplementary Table 3. Table showing the two-QTL model results for the top pairs with highest LOD score for full model for in vitro AM activity with pericarp color as an interactive covariate.

chr1	chr2	Pos1*	Pos2*	LOD_full+	LOD_fv1§	LOD_int¶	LOD_add†	LOD_av1¥	marker1	marker2	PVE (%)
1	4	25.62	53.98	11.30	6.29	4.29	7.01	1.99	01_25619342	04_53981655	25.7
1	6	25.85	45.29	9.74	4.72	0.04	9.70	4.68	01_25853452	06_45290650	22.7*
1	3	25.47	1.64	9.53	4.51	2.24	7.29	2.27	01_25471559	03_1635066	22.2
1	7	23.06	54.79	9.12	4.10	1.05	8.07	3.06	01_23064345	07_54788927	21.4
1	8	50.80	61.98	8.34	3.32	1.46	6.88	1.86	01_50802133	08_61977282	19.7
6	7	1.61	0.92	8.23	4.78	2.32	5.91	2.46	06_1610144	07_919333	19.5
1	10	25.85	60.25	8.17	3.16	1.64	6.53	1.51	01_25853452	10_60253536	19.4
1	2	54.28	63.45	7.64	2.62	1.04	6.60	1.59	01_54278686	02_63452353	18.2
3	6	3.40	45.20	7.45	4.00	1.16	6.29	2.84	03_3400572	06_45203134	17.9*
4	6	56.05	45.51	6.90	3.45	0.70	6.20	2.75	04_56051636	06_45505585	16.7
6	10	45.20	49.88	6.75	3.30	1.92	4.82	1.37	06_45203134	10_49881884	16.4
3	4	59.18	26.90	6.18	3.66	1.52	4.66	2.14	03_59183643	04_26897704	14.9
2	6	63.74	45.51	6.07	2.62	0.13	5.95	2.49	02_63735928	06_45505585	14.9*
2	3	61.15	3.51	5.80	3.28	1.40	4.40	1.88	02_61154136	03_3512414	14.3

*Position of the first and second locus (in Mb) in the full model
+LOD score for the full model (additive + interaction)
§LOD score for comparing full model vs. model with only the first locus
¶LOD score for the interaction effect alone (full model vs additive-only model)
†LOD score for the additive model
¥LOD score for comparing additive model vs. model with only the first locus (without interaction)
PVE (%): Percentage of Variance Explained by the full model having pericarp color as an interactive covariate.
*: the interaction term between loci was not significant at significance level of 0.05.

Supplementary Table 4. Table summarizing information of the three markers selected the were below the LOD threshold in the single QTL model of in vitro AM activity with pericarp color as a covariate. The table reports the marker name, chromosome (Chr), position (Pos) in Megabases, LOD score from the single QTL model and percentage of variance explained (PVE).

Marker	Chr	Pos (Mb)	LOD	PVE (%)
02_63735928	2	63.74	2.19	5.7
03_3400572	3	3.40	2.52	6.6
04_53981655	4	53.98	2.22	5.8

Supplementary Table 5. Table showing the four-loci model summary and drop one QTL at a time ANOVA for in vitro AM activity with Haley-Knott regression method and 175 observations.

Full model: y ~ 01_25.85 + Pericarp + 03_3.40 + 04_53.98 + 06_45.51 + 01_25.85* Pericarp + 01_25.85*04_53.98 + 06_45.51*Pericarp

	df	SS	MS	LOD	%var	*p*-value (χ^2)	*p*-value (F)
Model	8	8.09	1.01	16.57	35.34	2.70e-13	9.26e-13
Error	166	14.80	0.09				
Total	174	22.89					

Drop one QTL at a time ANOVA table

	df	Type III SS	LOD	%var	F-value	*p*-value (χ^2)	*p*-value (F)	Sig
01_25.85	3	4.16	9.41	18.18	15.55	0.000	5.81e-09	***
Pericarp	3	1.94	4.68	8.47	7.25	0.000	0.0001	***
03_3.40	1	0.38	0.97	1.67	4.27	0.035	0.0403	*
04_53.98	2	2.06	4.95	9.00	11.55	0.000	2.01e-05	***
06_45.51	2	1.98	4.77	8.65	11.11	0.000	2.97e-05	***
01_25.85* Pericarp	1	0.60	1.51	2.62	6.71	0.008	0.0104	*
01_25.85*04_53.98	1	1.74	4.21	7.59	19.47	0.000	1.83e-05	***
06_45.51*Pericarp	1	1.66	4.03	7.24	18.59	0.000	2.77e-05	***

df : degrees of freedom, SS: sum of squares, MS: mean square, %var: percentage of variance explained, Sig: significance at probability level of "*" 0.05, "**" 0.01 and "***" 0.001
